# In situ differentiation of iridophore crystallotypes underlies zebrafish stripe patterning

**DOI:** 10.1038/s41467-020-20088-1

**Published:** 2020-12-15

**Authors:** Dvir Gur, Emily J. Bain, Kory R. Johnson, Andy J. Aman, H. Amalia Pasolli, Jessica D. Flynn, Michael C. Allen, Dimitri D. Deheyn, Jennifer C. Lee, Jennifer Lippincott-Schwartz, David M. Parichy

**Affiliations:** 1 HHMI Janelia Research Campus, Ashburn, VA USA; 2 National Institute of Child Health and Human Development, NIH, Bethesda, MD USA; 3 Department of Biology, University of Virginia, Charlottesville, VA USA; 4 Department of Biology and Department of Cell Biology, University of Virginia, Charlottesville, VA USA; 5 Bioinformatics Section, National Institute of Neurological Disorder and Stroke, NIH, Bethesda, MD USA; 6 National Heart, Lung, and Blood Institute, NIH, Bethesda, MD USA; 7 Marine Biology Research Division, Scripps Institution of Oceanography, University of California, San Diego, La Jolla, CA USA

**Keywords:** Biophysics, Pattern formation

## Abstract

Skin color patterns are ubiquitous in nature, impact social behavior, predator avoidance, and protection from ultraviolet irradiation. A leading model system for vertebrate skin patterning is the zebrafish; its alternating blue stripes and yellow interstripes depend on light-reflecting cells called iridophores. It was suggested that the zebrafish’s color pattern arises from a single type of iridophore migrating differentially to stripes and interstripes. However, here we find that iridophores do not migrate between stripes and interstripes but instead differentiate and proliferate in-place, based on their micro-environment. RNA-sequencing analysis further reveals that stripe and interstripe iridophores have different transcriptomic states, while cryogenic-scanning-electron-microscopy and micro-X-ray diffraction identify different crystal-arrays architectures, indicating that stripe and interstripe iridophores are different cell types. Based on these results, we present an alternative model of skin patterning in zebrafish in which distinct iridophore crystallotypes containing specialized, physiologically responsive, organelles arise in stripe and interstripe by in-situ differentiation.

## Abstract (web summary)

The skin of zebrafish is patterned by alternating blue stripes and yellow interstripes which arises from guanine crystal-containing cells called iridophores that reflect light. Here the authors track iridophores and see that they do not migrate between stripes and interstripes, but instead differentiate and proliferate in place based on their micro-environment.

## Introduction

Biological patterning is ubiquitous in nature, but mechanisms underlying its establishment and maintenance have been well-documented in only a few instances that are unlikely to represent the full spectrum of pattern-forming systems^1,2^. Indeed, patterning can arise in response to graded positional information or by self-organization of interacting cells, and it can require alternative specification of cell types from a common progenitor or sorting out of cells that are heterogeneous already. Elucidating the mechanisms required to pattern cells in diverse tissues and organs is fundamental to understanding development and how phenotypes evolve.

**Fig. 1:**
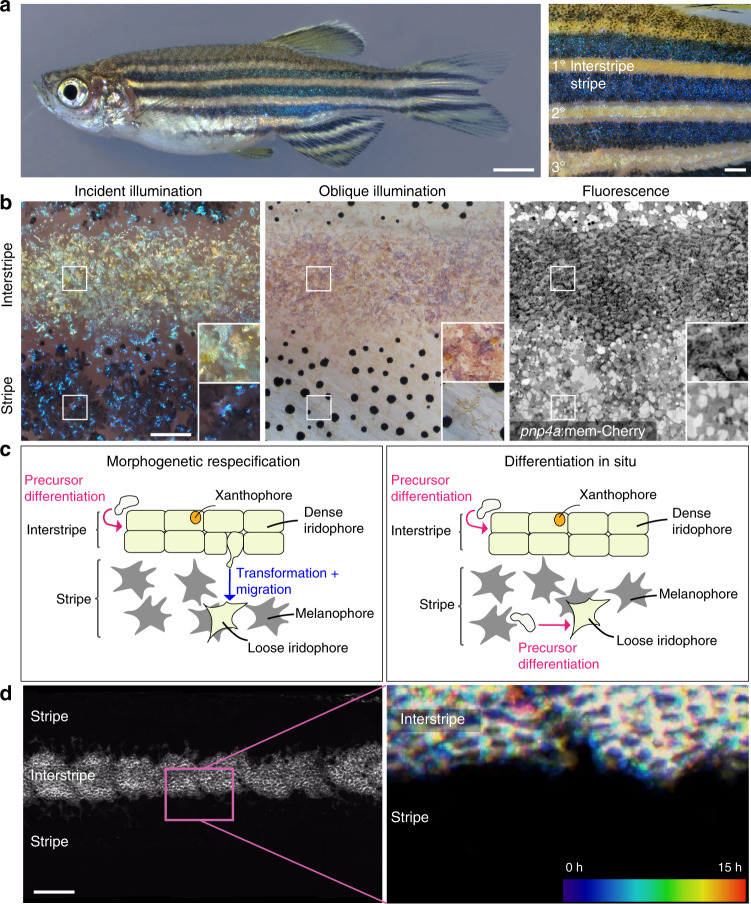
Anatomy, development, and models of zebrafish adult pigment patterning. **a** Left panel, an adult zebrafish showing light interstripes with intervening dark stripes. Right panel, a closeup showing the primary interstripe (1°) which develops first with stripes above and below, followed by secondary interstripes ventrally (2°) and dorsally with additional stripes, and ultimately a tertiary (3°) interstripe and stripe. **b** Closeups of first-forming 1° interstripe and stripes, illustrating overall pattern features, as well as morphologies and arrangements of iridophores. All panels are the same location in a single animal. Left panel is incident illumination showing iridescence of iridophore-reflecting platelets with yellowish tinge in the interstripe and bluish tinge in the stripe. Center panel is oblique illumination revealing surface features and non-iridescent colors of iridophores. Here, the fish has been treated with epinephrine to contract melanin granules of melanophores and pigment within xanthophores toward cell centers^26^, thereby better revealing iridophore morphologies. Right panel is membrane-targeted mCherry (mem-Cherry) driven at high levels in iridophores by regulatory elements of *purine nucleoside phosphorylase 4a* (*pnp4a*)^18,23,59^, revealing iridophore cell boundaries and arrangements. Pixel values are inverted for easier comparison to bright field images. Example shown is representative of >20 individual fish examined. **c** Two models for iridophore patterning in interstripes and stripes. In the morphogenetic respecification model (left panel), initially densely packed, cuboidal iridophores begin adopting a loose morphology as they and their progeny migrate out to populate the prospective stripe. In the differentiation in situ model (right panel), iridophores residing in interstripes and stripes are different cell types that have differentiated “in place” from a precursor population. Hence, loose iridophores in stripes are not lineally related to dense iridophores in interstripes. **d** The flank of a 7.5 standardized standard length (SSL)^24^ *pnp4a*:mem-Cherry fish. Left panel, fluorescence image showing the arrangement of labeled cells in the dense primary interstripe. Right panel, pseudo-temporal coloring representation of a 15 h time-lapse movie (zoomed to the region outlined in “**d**”) revealing that interstripe iridophores migrate primarily in the anteroposterior direction, with no apparent dorsoventral migration into the stripe region. Example image is representative of time-lapse videos from a total of 10 individuals during primary stripe formation at 7.0–7.5 SSL, as well as 15 individuals during secondary stripe formation at 10.0–12.0 SSL. Scale bars, **a** 2 mm, **b** 500 µm, **d** 500 µm.

The alternating dark (blue) and light (yellow) pigmented stripe pattern of adult zebrafish *Danio rerio* (Fig. 1a) is a useful model for dissecting patterning mechanisms^3–7^. Cells within the dark stripes include black pigment-containing melanophores; cells in the light stripes (known as “interstripes”) include orange pigment-containing xanthophores; and both dark stripes and light interstripes contain specialized cells called iridophores^8,9^. Iridophores are the major players for skin pattern establishment and reiteration in zebrafish. They behave as reflective cells, exhibiting angular-dependent changes in hue—iridescence—owing to membrane-bound reflecting platelets of crystalline guanine^9–11^. In the light interstripes, iridophores have a cuboidal shape and form an epithelial-like mat, presenting a “dense” morphological arrangement (Fig. 1b). In the dark stripes, by contrast, iridophores are sparse in number and stellate in shape, and are sometimes referred to as having a “loose” morphology^12^ (Fig. 1b). The iridophore’s importance in skin patterning has been demonstrated in experiments showing that genetically or experimentally induced deficiencies in iridophores cause pattern defects, including alterations in primary stripe positioning and boundary formation, and also lead to reductions or losses of secondary interstripes and stripes^13–17^. Likewise, an evolutionary truncation in iridophore development leads to an attenuated stripe pattern in the zebrafish relative *D. nigrofasciatus*^18^.

An elegant model explaining the iridophore’s role in stripe and interstripe formation links pattern establishment and reiteration to changes in iridophore morphology, proliferation, and migration^5,12,19^ (Fig. 1c, left panel). Densely arranged iridophores are proposed to first proliferate to fill the primary interstripe. Some of these cells then adopt a loose shape and migrate out into the stripe zone where they continue to proliferate. Subsequently, some loose iridophores reaggregate to adopt a dense morphology and thereby initiate secondary interstripes. The iridophore shape transitions from dense-to-loose and loose-to-dense are thought to resemble epithelial-to-mesenchymal transitions (EMT) and mesenchymal-to-epithelial transitions (MET), respectively. Signals by melanophores and xanthophores are proposed to determine the specific morphologies adopted by iridophores. Consistent with this idea, quantitative models incorporating dynamic morphological changes of individual iridophores are able to produce stripe patterning and robustness^20–22^.

A key prediction of the above model, hereafter referred to as “morphogenetic respecification,” is that some interstripe iridophores undergo an EMT-like transformation and migrate out from the interstripe zone to adopt a new, loose morphology and arrangement. In testing this prediction through a variety of approaches, we found that individual iridophores did not migrate out from the interstripe into the stripe. Instead, iridophores assumed a particular morphology at the time of their differentiation according to the presence or absence of melanophores, and this morphology remained fixed thereafter. We also observed that interstripe and stripe iridophores exhibited distinct organizations of guanine-reflecting platelets (i.e., crystal types) conferring intrinsic differences in color, and that only stripe-localized iridophores could modulate reflecting platelet spacings physiologically (blue → yellow). Furthermore, interstripe and stripe iridophores had distinct transcriptomic states. Based on these results, we propose an alternative new model for stripe pattern formation in the adult zebrafish, in which iridophore precursor cells undergo “differentiation in situ” into distinct iridophore types (i.e., crystallotypes; Fig. 1c, right panel). This process would depend on factors in the iridophore environment that impact the specification and subcellular organization of specialized organelles within iridophore precursors.

## Results

### Time-lapse imaging reveals iridophores do not migrate out from the interstripe

Stripe pattern establishment and reiteration in the zebrafish has been proposed to occur through morphogenetic respecification, in which iridophores differentiate to form a primary interstripe and then these cells or their progeny migrate out to contribute to stripes, as well as secondary interstripes and stripes^5,12,19,20^ (Fig. 1c, left panel). Individual cells would switch morphologies as appropriate to pattern context, undergoing morphogenetic respecification via processes resembling EMT or MET.

To test this model, we examined iridophore behaviors by time-lapse imaging of membrane-targeted mCherry (mem-Cherry) driven by regulatory elements of *pnp4a*^18,23^. If morphogenetic respecification accounts for variation in iridophore morphology and patterning, then events resembling EMT or MET should be observable. That is, densely packed iridophores of the completed interstripe should delaminate to populate the stripe, whereas loosely arranged iridophores of completed stripes should aggregate to initiate new interstripes. In over 300 h of recordings, we observed no instances in which interstripe iridophores—having the dense morphology—delaminated from their neighbors and assumed the loose morphology (Fig. 1d; 14,475 total cells, including 1637 cells located at interstripe edges). Likewise, interstripe iridophores that divided yielded daughter cells that remained in the interstripe (981 divisions, including 160 at interstripe edges; Supplementary Fig. 1a, and Supplementary Movies S1 and S2). These observations are inconsistent with the morphogenetic respecification model.

A different way to produce cells in distinct locations having distinct morphologies would be if iridophores populate interstripes and stripes by differentiating from a progenitor not yet specified to type (Fig. 1c, right panel). Iridophore morphology in this model would emerge by “differentiation in situ” in response to context-appropriate signals, and that same morphology would be retained thereafter by the cells or their progeny. A prediction of this model for iridophore patterning is that new cells should begin to express markers of iridophore differentiation during pattern formation. Consistent with this hypothesis, we frequently observed cells acquire or increase *pnp4a*:mCherry expression within developing stripes (Supplementary Fig. 1b–e and Supplementary Movies S2–S5).

Quantitative image analyses of proliferation and migration further supported pattern development by a mechanism of differentiation in situ. We found that proliferation of loose iridophores within stripes was greater than dense iridophores within interstripes, as noted previously^18^ (Supplementary Fig. 2a). Moreover, iridophores within interstripes tended to divide along an anterior–posterior plane, consistent with the known faster growth along this axis than dorsoventrally^24^ (Supplementary Figs. 1a and 2b). By contrast, planes of division by stripe iridophores were more uniformly distributed, in keeping with a rapid and relatively uniform occupancy of prospective stripe regions (Supplementary Figs. 1b–e and 2b). Dense iridophores also moved little, whereas loose iridophores could migrate up to several cell diameters and these movements tended to be biased away from the first interstripe (Supplementary Fig. 2c, d and Supplementary Movie S5).

### Fate-mapping and repeated imaging support a model of differentiation in situ

**Fig. 2:**
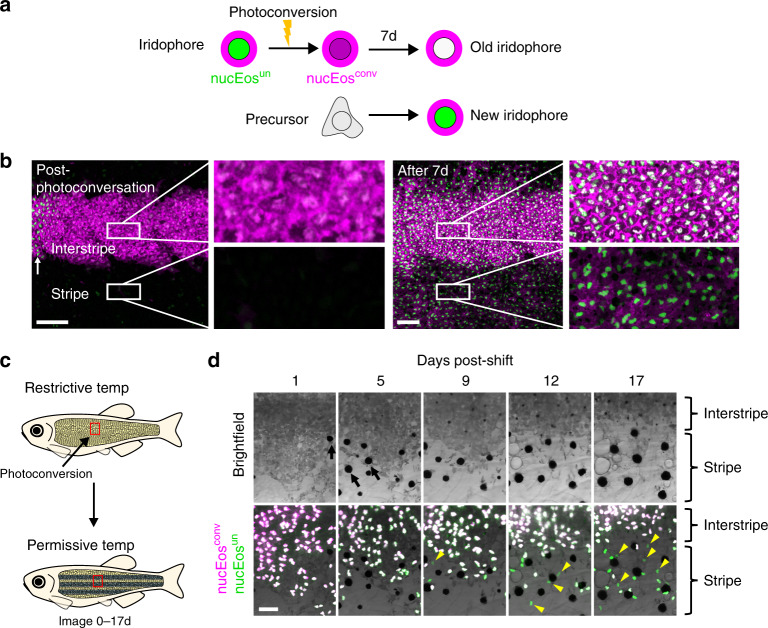
Photoconversion experiments to test models of pattern development and remodeling. **a** Fish were generated to have iridophores expressing nuclear-localizing Eos (nucEos^un^, green) and a membrane-targeted mCherry (mem-Cherry, magenta) driven by regulatory elements of *pnp4a*. Following photoconversion of an iridophore population, the converted nuclei will appear magenta (nucEos^con^). After 7 days, previously photoconverted nuclei will appear white (due to the combination of “new” green proteins and “old” magenta proteins), whereas nuclei of newly differentiated cells will appear green. **b** Tracking photoconverted iridophores in the interstripe revealed that stripe iridophores do not derive from the interstripe population. Following photoconversion of a region in the primary interstripe of a fish at 7.5 SSL, all nuclei appeared magenta (post-photoconversion), with surrounding mem-Cherry-labeled plasma membrane magenta-colored as well (left panel). After 7 days of additional development (8.6 SSL), at which time iridophores had populated the primary stripe, only nuclei with green signal were seen in the stripe zone, whereas interstripe nuclei were primarily white (right panel). Higher magnification images of boxed regions, show interstripe iridophores that retained nucEos^conv^, while also acquiring new nucEos^un^ (making their nuclei white; upper inset, right panel). Stripe iridophores, by contrast, lacked nucEos^conv^ and expressed only nucEos^un^ (making their nuclei green; lower inset, right panel). Example shown is representative of a total of eight individual fish examined. **c** Use of a temperature-sensitive *mitfa*^*vc7*^ allele to examine the effect of conditional melanophore development on iridophore pattern remodeling. For this experiment, iridophores were labeled only with a nuclear-localizing Eos (nucEos^un^, green; nucEos^conv^, magenta); after photoconversion nuclei appear magenta, or white as new nucEos^un^ was produced. **d** Brightfield (upper) and fluorescence superimposed on bright field (lower) following photoconversion and shift to permissive temperature to drive onset of melanophore differentiation. Iridophores labeled by nucEos expression were photoconverted at the beginning of the experiment and followed over 17 days to distinguish newly differentiating iridophores (green) from previously differentiated iridophores (white). As melanophores differentiated (see yellow arrows in top panel), the region of dense morphology iridophores receded dorsally. This change was accompanied by differentiation of new iridophores having green nuclei (see yellow arrowheads in bottom panel) in the newly forming stripe. Example shown is representative of a total of 12 individuals across two independent experiments. Scale bars, **b** 100 µm, **d** 50 µm.

We devised a further set of experiments to challenge the two models of iridophore patterning by following the long-term fates of iridophores marked by photoconversion (green → red) of nuclear-localizing *pnp4a*:nucEos fluorescent protein. In this experiment, photoconverted “old” iridophores will acquire white-colored nuclei over time due to their having both newly synthesized nucEos^un^ (unconverted green) and photoconverted nucEos^conv^ (red, here displayed as magenta) in their nuclei; by contrast, “new” iridophores developed from precursor cells will have only nucEos^un^ (green) in their nuclei (Fig. 2a)^25,26^. We reasoned that if individual iridophores change their morphological states and migrate out to contribute to both interstripes and stripes, as predicted by the morphogenetic respecification model, then marking cells in one pattern element should later yield marked cells in both pattern elements. On the other hand, if individual iridophores are fixed for their morphological state and contribute only to interstripes or stripes, as predicted by the differentiation in situ model, marked cells should retain their morphology and be confined to their original pattern element.

Immediately after photoconverting a region in the interstripe zone, all iridophores in this region had magenta nuclei, whereas iridophores in regions not targeted for photoconversion, including a very few loose iridophores already present in the stripe zone, had only green nuclei (Fig. 2b, post-photoconversion). After 7 days, only iridophores in the interstripe zone had white nuclei, whereas newly formed iridophores, having green nuclei (indicative of their acquiring *pnp4a* expression), could be seen mostly in the stripe zone (Fig. 2b, after 7 day). The presence of white-colored nuclei in the interstripe and their absence in the stripe indicates that interstripe marked cells did not migrate, favoring the model of differentiation in situ. In addition, we found that the formation of secondary interstripes was characterized by the development of cells newly expressing *pnp4a* within this region, suggesting differentiation with subsequent proliferation rather than active aggregation of widely dispersed cells^12^ (Supplementary Fig. 3).

The above analyses focused on a region in the middle of the flank. Because iridophore behaviors may differ between anatomical locations, we extended our analyses by examining distributions of *pnp4a*:mCherry+ cells in entire, individual fish imaged daily over 33 days. These analyses also revealed extensive differentiation of iridophores without indications of EMT (Supplementary Figs. 4 and 5a, b, and Supplementary Movie S6). In some instances, patches of dense iridophores anteriorly appeared to split between primary interstripe and ventral secondary interstripes, perhaps owing to rapid expansion of the flank directly over the swim bladder (Supplementary Fig. 5d and Supplementary Movie S7). In a minority of larvae (~20%), patches of five to ten closely associated iridophores developed anteriorly, dorsal to the primary interstripe. Cells in these patches sometimes maintained their tight associations and became incorporated into the secondary dorsal interstripe. In other instances, such cells were incorporated instead into the stripe (Supplementary Fig. 5c, e). Contrary to the expectations of the morphogenetic respecification model^12,20^, the few of these cells that transitioned from a nascent dense morphology to a loose morphology occurred already within prospective stripe regions. These observations highlight subtle region-specific differences in patterning events and suggest that, had state transitions occurred in a majority of cells or over a broader anatomical area, as predicted in the morphological respecification model, they should have been observed. That they were not observed lends further support to the model of differentiation in situ.

### Role of melanophores in iridophore pattern remodeling

Because melanophores reside in stripe zones but not interstripe zones, we wondered whether iridophore pattern remodeling (i.e., dense- versus loose-arrangement morphology) is impacted by melanophore presence. Prior work has hinted at this possibility as mutants for melanophore-inducing transcription factor (*mitfa*), which lack melanophores, have the dense morphology iridophores (characteristic of interstripe zones) over a broader area than wild-type fish^14,27^. To explore this further, we used a temperature-sensitive allele, *mitfa*^*vc7*^, that allows conditional differentiation or ablation of melanophores^26,28,29^ (Fig. 2c), and then examined the phenotypes of iridophores in different skin areas.

To assess whether iridophores in newly arising stripes, or regions newly devoid of stripes, were derived either from previously differentiated or newly differentiated cells, we marked cells by nucEos photoconversion, shifted fish between temperature regimes, and followed marked cells over time. When fish were shifted from restrictive temperature, in which they lacked melanophores, to permissive temperature, in which melanophore differentiation could occur, we found that preexisting, dense morphology iridophores receded, presumably owing to death, migration from the region, or both, and that new iridophores differentiated into a loose arrangement in regions where melanophore differentiation had occurred (Fig. 2d and Supplementary Fig. 6a). Reciprocal temperature shifts to ablate melanophores led to a similar loss of preexisting loose iridophores and the differentiation of new dense iridophores (Supplementary Fig. 6b). Though we cannot exclude the possibility that some preexisting iridophores were incorporated into remodeled pattern elements, these results suggest that the presence of melanophores has a major effect on the pattern remodeling of iridophores, specifically, in promoting a loose morphology.

### Distinct crystal morphology and ultrastructural organization, but shared chemistry of loose and dense iridophores

Differences in iridophore morphologies (dense/cuboidal versus loose/stellate) and our failure to observe transitions between these two states, raised the possibility that iridophores of dense/cuboidal morphology in interstripes and loose/stellate morphology in stripes represent distinct cell subtypes, analogous to neuronal subtypes^30^. To test this possibility, we evaluated the subcellular architecture, physiology, and gene expression of dense/cuboidal iridophores in stripes versus loose loose/stellate iridophores in interstripes.

**Fig. 3:**
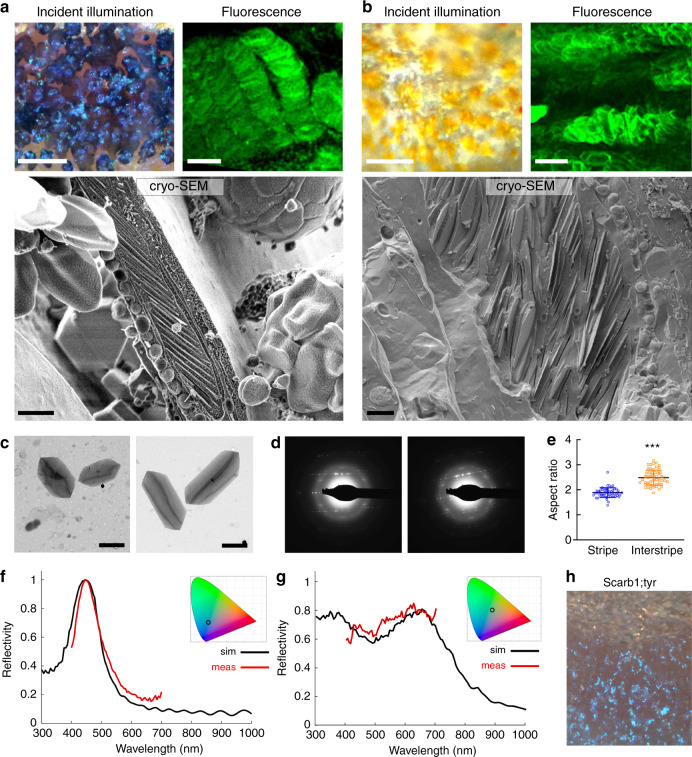
Loose versus dense iridophores have distinct crystal morphologies and ultrastructural organizations, but shared chemistry. **a** Loose iridophores in stripe region viewed by incident illumination, fluorescence, and high pressure–frozen, freeze-fractured cryo-SEM (Cryo-SEM). The incident illumination image shows blue iridophores on top of black melanophores; the fluorescent image reveals malachite green (MG) labeled iridophores (pseudo colored green) with highly ordered arrays of guanine crystals; and the Cryo-SEM image shows iridophore cytoplasm with highly disordered arrays of crystals. *n* = 5 adult fish for incident illumination and fluorescence, and *n* = 4 adult fish for cryo-SEM. **b** Dense iridophores in interstripe region viewed by incident illumination, fluorescence, and Cryo-SEM. The incident illumination image shows silvery iridophores covered by yellow xanthophores; the fluorescent image reveals MG-labeled iridophores with disordered arrays of guanine crystals (pseudo colored green); and the high pressure–frozen, freeze-fractured cryo-SEM micrograph shows iridophore cytoplasm with disordered arrangements of crystals. *n* = 5 adult fish for incident illumination and fluorescence, and *n* = 4 adult fish for cryo-SEM. **c**–**e** TEM analysis of crystals isolated from iridophores from either the stripe or the interstripe regions of adult fish (*n* = 4 adult fish). **c** TEM micrographs of crystals isolated from stripe (left panel) and interstripe regions (right panel); **d** TEM-based electron diffraction of the crystals shown in Supplementary Fig. 7, isolated from stripe iridophores (left panel) and interstripe iridophores (right panel); **e** graph of aspect ratio (length/width) of stripe iridophores (blue) and interstripe iridophores (orange), *n* = 60 for stripe isolated crystals and *n* = 57 for interstripe isolated crystals, Data are represented as mean ± SEM. The *p* value, *p* < 0.0001, was determined using two-tailed Mann–Whitney test. **f** Simulated reflection (black) and measured reflection (red) from a stripe iridophore. **g** Simulated reflection (black) and measured reflection (red) from an interstripe iridophore. Insets in both **f** and **g** show the corresponding reflectance color on a CIE (International Commission on Illumination) chromaticity space diagram. **h** Incident illumination image of an adult fish lacking melanin in melanophores and carotenoids in xanthophores due to mutations in *tyrosinase* and *scarb1*, respectively. The image shows iridophore-type-specific coloration is independent of melanin and carotenoids, consistent with reflectance data obtained for stripe (**f**) and interstipe iridophores (**g**). Scale bars, **a**, **b** (left panels) 50 μm, **a**, **b** (right panels) 4 μm, **a**, **b** (bottom panels) 1.5 μm, **c** 2 μm.

Because iridophores depend for their iridescence on stacks of membrane-bound reflecting platelets consisting of crystalline guanine^4,11^, we first asked whether numbers, sizes, or arrangements of these crystals differ between iridophores found in interstripe versus stripe regions. To visualize guanine crystals in situ required a reagent that would adhere to guanine crystals, and so we screened 12 cell-permeable dyes, chosen for their ability to form both hydrogen bonds and pi-stacking interactions^31^. We found that malachite green efficiently bound guanine and therefore used it to examine guanine crystal organization in iridophores from interstripe versus stripe regions. Incident illumination images of the stripe zone showed blue, loosely distributed iridophores on top of black melanophores, whereas images of the interstripe zone showed dense silvery iridophores covered by yellow xanthophores (Fig. 3a, b, incident illumination). Notably, malachite green labeling of guanine crystals within iridophores in these two zones revealed remarkably stacked arrays of crystals in loose iridophores from the stripe, but markedly disordered arrays of crystals in dense iridophores from the interstripe (Fig. 3a, b, upper panels).

To assess the ultrastructural organization of iridophores under near-physiological conditions, in which crystal organization and cytoplasmic spacing are likely to be retained, we used cryogenic scanning electron microscopy (cryo-SEM). Crystal arrays of loose iridophores from stripes were remarkably ordered, with 20–30 layers of parallel crystals having an average thickness of 27 ± 7 nm (*n* = 82), neatly separated by thin layers of cytoplasm of average thickness 131 ± 24 nm (*n* = 91; Fig. 3a, lower panel). By contrast, crystal arrays of dense iridophores from interstripes were disordered, varying in both orientations and spacings between crystals (Fig. 3b, lower panel), with 30–40 crystals per cell, and a similar average crystal thickness of 25 ± 8 nm (*n* = 130) and an average cytoplasm spacing of 186 ± 81 (*n* = 145).

Beyond differences in crystal arrangements, the shapes and sizes of crystals appeared to differ between loose iridophores in stripes and dense iridophores in interstripes. To quantify these differences, we isolated skin separately from stripes (Fig. 3c, left panel) and interstripes (Fig. 3c, right panel), and extracted crystals for transmission electron microscopy (TEM) and electron diffraction analyses. While the crystals in cells from both tissue regions comprised plates of β-guanine (Fig. 3d and Supplementary Fig. 7), crystals isolated from stripe iridophores were smaller (3.9 ± 0.4 versus. 5.3 ± 0.9 µm; *n* = 60) and had smaller aspect ratios than crystals from interstripe iridophores (1.9 ± 0.2 versus 2.5 ± 0.3 µm; *n* = 57; Fig. 3e). In situ Raman spectroscopy of individual cells further validated that crystals in loose iridophores in stripe zones and dense iridophores in interstripe zones consist of β-guanine, and failed, within the accuracy afforded by these measurements, to reveal other components, suggesting that differences in crystal morphology are not related to their chemistry (Supplementary Fig. 8).

### Iridophore subtypes differ in their optical properties

Differences in colors reflected by stripe iridophores (blue) and interstripe iridophores (silvery-yellow) have sometimes been ascribed to influences of pigments contained within melanophores and xanthophores, respectively^5,32^. Given the differences we observed in reflecting platelet architectures of iridophores from stripes versus interstripes, however, we reasoned that reflected spectra might be intrinsic properties of iridophore subtypes. Consistent with this hypothesis, we found close matches between spectra predicted from simulations based on a Monte Carlo transfer matrix^33^ (with morphometic data derived from cryo-SEM) and empirical reflectance spectra recorded for individual cells by hyperspectral imaging microscopy^34^ (Fig. 3f, g). Indeed, simulations for ordered-crystal iridophores from stripes predicted a peak in the blue region at 450 nm approaching unity reflection, whereas simulations for disordered-crystal iridophores from interstripes predicted a broad wavelength reflection. In addition, while the reflection from the ordered-crystal iridophores was highly dependent on the angle of incident light, the reflection from disordered-crystal iridophores was not (Supplementary Fig. 9).

We further found that intrinsic differences in iridophore optical properties could generate a strong contrast in color between stripes and interstripes independent of pigments in other cell types. This was manifested in fish that lacked both melanin in melanophores and carotenoids in xanthophores, owing to mutations in *tyrosinase* and *scarb1*, respectively^23^. Here, differences in color between stripes and interstripes (i.e., blue versus silvery-yellow) persisted even in the absence of other pigments (Fig. 3h). Together, these results demonstrate the intrinsic differences in optical properties between ordered-crystal iridophores of stripes and disordered-crystal iridophores from interstripes.

### Disordered-crystal and ordered-crystal-containing iridophores remain distinct throughout development

**Fig. 4:**
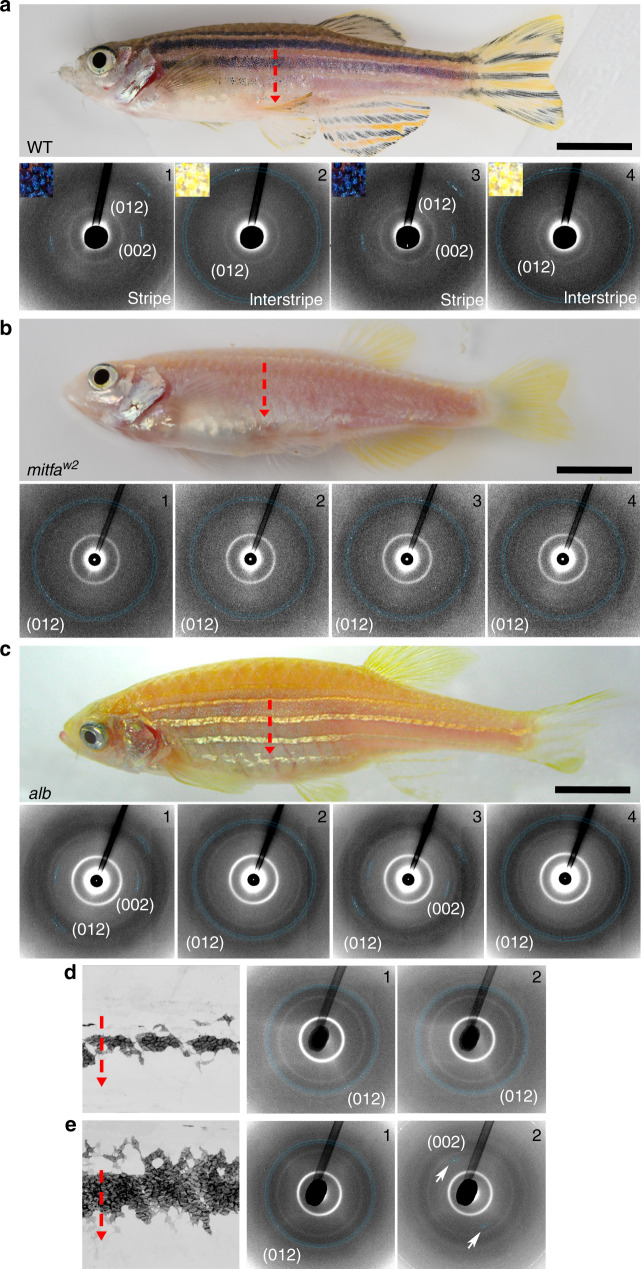
Disordered- and ordered-crystal-containing iridophores remain distinct throughout development. **a** Upper panel shows wild-type zebrafish with the red vertical dotted arrow showing, where X-ray diffraction measurements were made. Lower panels 1 through 4 show X-ray diffraction pattern measurements in stripe and interstripe regions, with upper left insets showing the incident illumination differences in these regions. Diffraction patterns collected in the stripe regions (1 and 3) had low-angular distributions with a punctuated-like signal, indicating iridophore crystals in these regions are parallel to one another. Diffraction patterns collected in the interstripe regions (2 and 4), by contrast, exhibited high-angular distributions with a full-ring signal, indicating iridophore crystals in these regions are not well aligned. *n* = 3 different fish. **b** X-ray diffraction measurements as in **a** made in *mitfa*^*w2*^ mutant fish, using a vertical line scan across the trunk of the fish. The typical diffraction pattern of the ordered stripe iridophore is missing in this line scan, and the observed diffractions are of high-angular distribution (“full ring”). *n* = 3 different fish. **c** X-ray diffraction measurements as in **a** from *albino* mutant (*alb*). The overall diffraction pattern resembles that of wild-type fish, with highly ordered diffraction patterns of the (002) and (012) diffraction planes throughout the stripe regions (1 and 3), and high-angular distribution of only the (012) diffraction plane throughout the interstripe regions (2 and 4). *n* = 3 different fish. **d**, **e** X-ray diffraction patterns from vertical lines measured across the trunk of ~6 SSL (**d**) and ~6.9 SSL (**e**) wild-type zebrafish. Left panels, illustrate representative individuals and iridophore patterns from repeated image series (e.g., Supplementary Fig. 4). Panels 1 and 2 show X-ray diffraction patterns from areas in the 1° interstripe and adjacent to the 1° interstripe, respectively. In **d**, both regions show a high-angular distribution of the (012) diffraction plane. In **e**, a low-angular distribution diffraction of the (002) plane (2) is visible (white arrows) just adjacent to the first interstripe region (1). *n* = *3* different fish. Scale bars, **a**–**c** 4 mm.

To map the structural organization of iridophores across the entire skin pattern, we used synchrotron-based micro X-ray diffraction, which allows large areas to be scanned, while still providing information on orientations and anisotropy of crystal arrays at the level of a single cell. In this system, high-angular distribution diffractions having a full-ring signal are indicative of crystal orientations that vary (i.e., disordered), whereas low-angular distribution diffractions having a punctuated-ring signal are indicative of crystals that are consistently oriented (i.e., ordered)^10^. Dorsoventral line scanning across the flank of the fish demonstrated there were consistent differences in structural organization of stripe versus interstripe regions (Fig. 4a). Specifically, based on their (012) and (002) diffraction planes^10,35^, crystal plates in iridophores of the stripe zone were well oriented (i.e., ordered; Fig. 4a, panels 1 and 3, and Supplementary Fig. 10), whereas those in the interstripe zone were nonaligned (i.e., disordered; Fig. 4a, panels 2 and 4, and Supplementary Fig. 10).

Our *mitfa*^*vc7*^ photoconversion results (see Fig. 2c) raised the possibility that melanophores promote the differentiation of progenitors into iridophores with ordered-crystal arrays. We tested this idea using micro X-ray diffraction to evaluate the crystals architecture in iridophores of two zebrafish mutants: null-allele *mitfa*^*w2*^ and *albino*. In *mitfa*^*w2*^ mutants, melanophores are missing owing to a defect in their specification; in *albino* mutants, melanophores are present but lack melanin^27,36^. We reasoned that if melanophores drive iridophore differentiation toward the ordered crystallotype, then *mitfa*^*w2*^ mutants should be deficient in iridophores having ordered crystals, whereas *albino* mutants should retain ordered iridophores, similar to the wild type. Line scans across the flanks of *mitfa*^*w2*^ fish revealed mostly high-angular distribution (012) diffractions, typical of the disordered crystallotype (Fig. 4b and Supplementary Fig. 11). Scanning the entire fish showed some diffraction patterns corresponding to ordered iridophores, but these were located toward the posterior and were a minor component of the diffractions (Supplementary Fig. 12). The same analysis on *albino* fish revealed alternating diffraction patterns similar to that seen in wild type (Fig. 4c and Supplementary Fig. 13). These results suggest that melanophores enhance the differentiation of ordered-crystallotype iridophores.

We next examined the relative developmental timing of precursor differentiation into disordered and ordered crystallotypes by assessing micro X-ray diffraction patterns over ontogeny. In fish of 6.0 mm standardized standard length (SSL) and 6.5 SSL, which have only a single interstripe and very few adult melanophores^24^, we observed only disordered-crystallotype iridophores (having high-angular distribution diffraction patterns of the (012) plane; Fig. 4d, and Supplementary Figs. 14 and 15). When fish were ~6.9 SSL, with a substantial complement of melanophores and loose iridophores, low-angular distribution diffraction patterns of the (002) plane, typical of ordered-crystallotype iridophores, became visible (Fig. 4e, see white arrows, and Supplementary Fig. 16). These results supported the idea that precursor cells differentiate into ordered-crystallotype iridophores only after the differentiation of disordered-crystallotype iridophores and in the presence of melanophores.

### Ordered and disordered crystallotypes exhibit distinct transcriptomic signatures

**Fig. 5:**
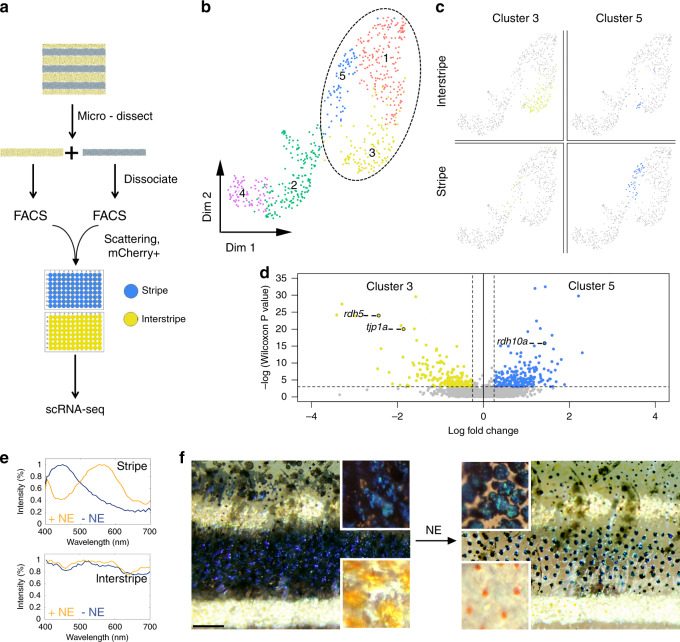
Disordered- and ordered-crystal-containing iridophores exhibit distinct transcriptomic signatures and response to stimuli. **a** Experimental design of single-cell RNA-sequencing experiment. **b** Two-dimensional UMAP representation of the collected skin cell clusters (dashed ellipse marks iridophores). **c** Anatomical origin (stripe versus interstripe) of iridophores from clusters 5 (blue) and 3 (yellow). **d** A volcano representation of differentially expressed genes between clusters 5 (blue) and 3 (yellow), where 192 genes were upregulated in cluster 5 and 158 were upregulated in cluster 3. **e**, **f** The response of an adult zebrafish skin pattern to norepinephrine (NE) stimulation. **e** The optical response of individual iridophores from the stripe (upper panel) and interstripe (lower panel). In the stripe, the reflection peak of an ordered iridophore shifts from ~450 to ~570 nm upon NE treatment. In the interstripe, only minor changes in the reflection spectra occur in response to NE. *n* = 4 different fish (**f**) illustrates optical response from relaxed, untreated fish (−NE) to the treated fish (+NE). Note the differences between the blue stripe and the two-flanking yellow interstripes and how this changed with NE treatment. Before treatment, a deep blue color for the stripe region and a golden-yellow color for the interstripe region is observed. After NE treatment, the contrast between the stripe and the interstripe is drastically reduced. This color change arises because NE causes pigment granules within the melanophores in the stripe to aggregate in the cell center and blue iridophore reflectance to shift from a dark-blue to green-yellow hue (see upper insets), while in the interstripe, NE causes the pigment granules within the xanthophores to aggregate and silvery iridophores to have only a minor color change (lower insets), *n* = 3 different fish. Scale bar, **f** 400 μm.

We next tested whether stripe and interstripe iridophores also have distinct transcriptomic signatures by single-cell RNA-sequencing (scRNA-seq; Fig. 5a). Dimensionality reduction followed by unsupervised clustering revealed five clusters (Fig. 5b, Supporting file—Table 1), three of which (clusters c1, c3, and c5) contained cells expressing high levels of known markers of iridophores (i.e., *gpnmb*, *pnp4a*)^37^ (Supplementary Figs. 17 and  18a, b), as well as genes that were recently identified as iridophore markers (i.e., *alx4a*, *alx4b*)^23^ (Supplementary Figs. 17 and  18c, d). The expression levels of these iridophore markers, as well as other markers for pigment cells, were lower in clusters c2 and c4 (Supplementary Fig. 17), suggesting these clusters potentially represent cells that are not fully differentiated.

Next, we tested whether any of the iridophore clusters recovered by scRNA-seq were originated specifically from either the stripe or the interstipe locations. We found that cluster c5 was mostly stripe specific, as 85% of cells in cluster c5 originated from stripes, whereas cluster c3 was mostly interstripe specific, as 84% of cells in cluster c3 originated from interstripes (Fig. 5c). Cells of the third iridophores cluster, c1, were shared between the interstripe (44%) and stripe (56%; Supplementary Fig. 19). Several hundred loci were differentially expressed between cells of clusters c3 and c5 (Fig. 5d and Supporting file—Table 2), highlighting candidate genes that may contribute to structural or other differences between stripe and interstripe iridophores. For example, it was recently shown using immuno-labeling that Tight Junction Protein 1a (tjp1a) is highly expressed only in dense iridophores^19^ (found in the interstripe), and that knocking out *tjp1a* results in the disruption of the stripe pattern due to dense (interstripe) iridophores invading the stripe regions^19^. In line with these findings, we found that *tjp1a* is highly expressed in cluster c3 (mostly interstripe iridophores) and expressed at lower levels in cluster c5 (mostly stripe iridophores). Other interesting genes that were differentially expressed at high levels were *retinol dehydrogenase 5* (*rdh5*) and *retinol dehydrogenase 10a* (*rdh10a*), upregulated in cluster c3 and cluster c5, respectively. These genes code for proteins that catalyze key oxidation–reduction reactions in the visual cycle, and could be related to the presence of visual pigments in these cells and their reported ability to sense light^38^.

We also performed a gene-set enrichment analysis of the differentially expressed genes using FishEnrichr^39,40^ (Supplementary Table 1 and Supplementary Table 2). The gene enrichment analysis highlighted many differentially regulated pathways, including responses to purine-containing compounds, and cAMP pathways (Supplementary Table 1), which were both upregulated in cluster c5 (mostly stripe iridophores) and might be involved in iridophore environmental sensing and reaction to specific stimuli (see below). Another set of differentially enriched pathways were related to the cytoskeleton, including pathways for all three main cytoskeleton components, actin filaments, intermediate filaments, and microtubules (Supplementary Table 2), consistent with roles in mediating differences in structural organization and crystal array architectures between stripe and interstripe iridophores. We concluded from these analyses that iridophores of interstripe and stripe zones are transcriptionally distinct.

### Physiological color change differs between iridophore subtypes

Color pattern can be influenced physiologically^41,42^, as some types of pigment cells disperse or contract pigment granules in response to endocrine and neuroendocrine factors, including norephinephrine (NE)^43–45^. We wondered whether morphological differences between iridophore subtypes might be accompanied by physiological differences as well. To test this, we bathed isolated fish skin in 10 µM NE solution. Upon NE treatment, stripe-derived iridophores exhibited a ~120 nm shift in peak reflectance, whereas interstripe-derived iridophores did not exhibit gross changes in reflected light spectra, measured by hyperspectral imaging (Fig. 5e). Viewing the response to NE in the context of the whole tissue also revealed a reduction in the color differences between the stripe and the interstripe locations (Fig. 5f). These results support prior work showing that iridophores of different shapes respond differently to NE^43^, and further demonstrate that only stripe iridophores change their color upon NE treatment. This differential response, in conjunction with the known aggregation of granules within melanophores and xanthophores^44,45^, could contribute to the dramatic reduction in contrast between stripe and interstripe zones under NE treatment, which causes the prominent zebrafish stripe pattern to diminish (Supplementary Movie 8).

## Discussion

Pigmentation of teleost fish has become a valuable system for understanding pattern formation in animals, including how changes in pattern-forming mechanisms lead to phenotypic variation within and between species^3,5,6^. In zebrafish, a widely accepted model links dynamic changes in iridophore shape—between a dense morphology in interstripes and a loose morphology in stripes—to establishment and reiteration of pattern^12,19,20,22^. In this model, iridophores of interstripes and stripes are similar cells that have adopted different morphologies, as they migrate into different regions. Using diverse tools, we found no evidence for morphological transitions by individual iridophores. Rather, our data support an alternative model of differentiation in situ for how the reiterated stripe pattern of zebrafish develops. In this model, iridophore precursors in developing stripes and interstripes differentiate based on their microenvironment into distinct iridophore crystallotypes, with different subcellular organization and physiological responsiveness.

We found several structural disparities between iridophores of interstripes and stripes; iridophores of interstripes had larger reflecting crystal platelets that were disordered, whereas those of stripes had smaller crystal platelets that were uniformly stacked and oriented. The colors of these cells differed as well: iridophores in interstripes were silvery-yellowish, and iridophores in stripes were blue, and these differences were autonomous properties of the cells, not a consequence of pigments contained in other pigment cells with which iridophores associate. Physiological responses also differed: disordered-crystal platelets of interstripe iridophores were refractory to NE, whereas, ordered-crystal platelets of stripe iridophores changed their cellular organization upon NE treatment. Finally, iridophores from interstripes and stripes had distinct gene expression profiles.

We found no evidence that individual iridophores undergo state transitions, as would be expected if cells originating in one pattern element disperse to populate another. Photolabeled iridophores observed over short or long periods failed to migrate between interstripe and stripe zones, even when they were challenged to undergo transitions in the context of pattern remodeling (stimulated by changes in melanophore abundance). Instead, development and remodeling of interstripes and stripes involved de novo differentiation, subsequent proliferation, and in some cases migration of iridophores with morphologies appropriate to their location within the pattern. Only in a minority of fish, and in a small anatomical region, did we observe patches of initially dense iridophores assume a loose arrangement. Such behaviors occurred within prospective stripes, rather than at boundaries between interstripes and stripes, as previously postulated^5,12^, and involved cells that had not yet fully differentiated.

The apparent conflict between our observations and those of prior studies^5,12,19^ may reflect differences in how iridophores and their progenitors have been visualized. Previous analyses used a *sox10:CreER*^*t2*^ transgene that can mark most or all postembryonic neural crest derivatives, including multipotent progenitors in the peripheral nervous system, and cells transiting from the peripheral nervous system to the hypodermis of the skin^12,46^. In this case, clonal spread over the flank from interstripe-to-stripe, and stripe-to-interstripe, is indeed apparent as progeny of individual progenitors migrate to the skin or expand their numbers at the hypodermis. If individual cells appear to switch between dense and loose morphologies, these may represent iridophore precursors transiently associated with one pattern element or another, but not yet committed to crystallotype. By contrast our analyses employed *pnp4a* transgenes, expressed as iridophores begin to differentiate and our photoconversion approach, tracking individual *pnp4a* expressing cells, allowed us to map the fate of differentiated iridophores during development. We postulate that *pnp4a*-labeling allowed us to distinguish later stage, committed iridophores or iridoblasts from earlier stage iridoblasts or earlier progenitors that had not yet become fate restricted.

These data all point to a model of stripe patterning that depends on differentiation in situ. In this model, latent progenitors associated with the peripheral nervous system that have transited to the skin during the larva-to-adult transformation^3^ expand clonally as early iridoblasts—not specified to subtype—and subsequently differentiate according to cues in the microenvironment they encounter. Our data, together with those of others, suggest that some of these signals depend on melanophores^14,15,25,47^, promoting differentiation of iridoblasts toward a state having ordered reflecting crystal platelets that are physiologically responsive (blue ⟷ yellow) in stripes, and away from an alternative state of disordered-crystal platelets lacking physiological responsiveness (silvery-yellow) in interstripes. Additional signals from xanthophores, iridophores, and other cell types likely contribute as well. Whether these events of specification unfold as iridoblasts expand their territory within the plane of the skin hypodermis, or as they arrive at the hypodermis after migrating from progenitor niches within the peripheral nervous system, remains to be determined. Whichever mode of iridoblast morphogenesis holds true, our findings highlight the importance of extrinsic factors that specify and promote the in situ differentiation of iridophore subtypes, during pattern establishment and reiteration. The resulting iridophore subtypes likely allow the zebrafish to alter its skin patterning to make it more or less distinctive, a trait crucial for the fish to be able to join shoals or obscure itself^48,49^.

## Methods

### Fish stocks, rearing conditions, transgenic line production, and CRISPR/Cas9 mutagenesis

We reared zebrafish under standard conditions (14 L:10 D at ~28 ˚C) except as indicated and in accordance to HHMI-JFRC ethical committee via animal Care and Use protocol 16-137 and UVA IACUC protocol 4170. Staging followed^24^. Stocks were wild type WT(ABb), a derivative of AB^wp^; Tg(*pnp4a:palm-mcherry*)^*wprt10Tg*^ expressing membrane-targeted mCherry^18,50^; *mitfa*^*w2*^^27^; temperature-sensitive *mitfa*^*vc7*^^29,51^ raised at permissive temperatures (22 °C) or restrictive temperature (30 °C); *scarb1*^*vp32rc1*^^52^; and *albino*^*b4*^ (*slc45a2*). To generate Tg(*pnp4a-nucEos*)^*wprt30Tg*^, we subcloned ~8 kb from a recombineered BAC used for constructing Tg(*pnp4a:palm-mCherry*) and replaced the original fluorophore with nuclear-localizing multimeric EosFP^25,53^ by restriction/ligation cloning. We injected this construct into single-cell embryos along with *Tol2* transposase mRNA using standard methods^54^, and screened for germline incorporation by transgene fluorescence. To generate fish that lacked both carotenoid pigmentation and melanin, we injected *scarb1* mutants with 500 ng/μl Cas9 protein (PNA Bio) and two T7-transcribed guide RNAs targeting *tyrosinase* (*tyr*; GGGCCGCAGTATCCTCACTC, GGCGTTTCTGCCTTGGCATC) at 200 ng/μl each.

#### Imaging

We acquired images on Zeiss Axio Observer inverted microscopes equipped with Axiocam HR or Axiocam 506 color cameras, a Yokogawa CSU-X1M5000 laser spinning disk with Photometrics Evolve or Hamamatsu ORCA-Flash 4.0 cameras, an AxioZoom v16 stereomicroscope with Axiocam 506 color camera, or Zeiss LSM 800 scanning laser confocal with GaAsP and Airyscan detectors, all running ZEN blue software. For repeated daily imaging, we raised individuals in separate beakers. They were anesthetized briefly, imaged, and allowed to recover.

#### Photoconversion

We photoconverted fish expressing *pnp4a:nucEos* stably or mosaically using a Zeiss LSM 800 scanning laser confocal with a 405 nm laser and ZEN blue software. When we photoconverted entire flanks (Supplementary Fig. 3), we placed fish in a box lined with aluminum foil and exposed them to full intensity output from a Zeiss HXP 120 V compact light source.

#### Time-lapse imaging and analyses

Pigment cells were imaged ex vivo in their tissue environment on a laser spinning disk microscope (above)^50^. We imaged the entire flank between gut and caudal fin peduncle of Tg(*pnp4a:mCherry*) fish every 5 min for 15 h, unless otherwise noted. To capture rare behaviors over large areas, images were typically collected as multiple tiles for each specimen, then stitched computationally using Zeiss ZEN software. Supplemental Movies S1–S5 illustrate regions of interest from larger views. Analyses focused on the area including the primary interstripe, ventral primary stripe, and ventral secondary interstripe (where applicable). All iridophores in these regions were included in proliferation and migration analyses. Proliferating iridophores were evident as single cells that rounded up and then divided to generate adjacent daughter cells. We measured the plane of proliferation using the angle tool in ImageJ from the center of each daughter cell in the frame immediately following cytokinesis. To analyze migration, we used ImageJ to determine start and end points of all stripe-associated iridophores that moved ≥1 cell diameter over the course of each time-lapse movie. Trunks were examined from fish at stages 7.0 SSL (*N* = 5), 7.5 SSL (*N* = 5), 10.0 SSL (*N* = 11), and 12.0 SSL (*N* = 4) were included in cell behavioral analyses. Iridophores were classified as dense (surrounded by other iridophores, bright mCherry expression on all sides), loose (stellate, dim mCherry expression), or edge (contacting other dense iridophores over ~75% of the cell perimeter with the remainder open to the prospective stripe region). Total cells analyzed were: 7.0 SSL—630 dense, 57 loose, 359 edge; 7.5 SSL—1230 dense, 338 loose, 423 edge; 10.0 SSL—4113 dense, 2047 loose, 523480 edge; 12.0 SSL—1657 dense, 1508 loose, 186 edge.

#### Temperature shift experiments

We injected *mitfa*^*vc7*^; Tg(*pnp4a:mCherry*) with *pnp4a:nucEos* and *Tol2* mRNA using standard methods. We raised the fish at restrictive temperature (30 °C) or permissive temperature (22 °C) until the primary interstripe, primary stripes, and ventral secondary interstripe developed at permissive temperature. We then photoconverted mosaically expressed patches of *pnp4a*:nucEos as described above, and shifted fish to the opposite temperature.

#### Synchrotron-based micro wide X-ray diffraction

Fish (larvae or adults) were euthanized in MS222 (E10521, Sigma-Aldrich) and were immersed in a physiological buffer (PBS, P3813, Sigma-Aldrich,136 mOsm/kg). For adult fish, fresh skin sections were obtained and mounted on a lead tape between two kapton windows. For larvae fish, entire freshly euthanized fish were mounted on a lead tape between two kapton windows. In situ wide X-ray diffraction (WAXD) was obtained at the μ-Spot beamline, in the synchrotron radiation facility BESSY II, Helmholtz-Zentrum Berlin für Materialien und Energie, Berlin, Germany. Samples were mounted on a *y*–*z* scanning table and scans of the sample in areas of interest were performed. The microbeam was defined by a toroidal mirror and a pinhole of either 10 or 30 μm diameter close to the sample, providing a beam size of ~10 × 10 μm^2^ or 30 × 30 μm^2^ at the sample position. Line scans were performed across the flank of the fish, with two to four data points collected for each interstripe, and three to six data points collected for each stripe. An energy of 15 keV (*λ* = 0.82656 Å) was selected by a Mo/BC multilayer monochromator. The 2D SAXS/WAXD patterns were measured by using a MarMosaic 225 CCD-based area detector (Rayonix) placed at a sample–detector distance of 286 mm. The beam center in the detector and the sample–detector distance were calibrated, using a powder X-ray diffraction pattern of synthetic guanine powder standard (Sigma-Aldrich). Radial integration of the 2D scattering patterns was performed using and DPDAK^55^. The data were normalized with respect to the primary beam monitor (ionization chamber) and corrected for background caused by pinhole and air scattering.

#### Hyperspectral imaging

Fish were euthanized in MS222 (E10521, Sigma-Aldrich), then relevant skin portions were dissected and placed in PBS solution (P3813, Sigma-Aldrich) at room temperature. Spectral measurements were performed using a PARISS® hyperspectral imaging system (Lightform Inc. with PARISS software v1.14)), which acquires instantaneously 380–980 nm spectra from each pixel of a line of pixels when pixel size was 1.25 × 1.25 μm^2^. The hyperspectral imager was mounted on a Nikon Eclipse 80i microscope. Spectral calibration was performed using a MIDL® Hg^+^/Ar^+^ wavelength calibration lamp (Lightform Inc.) with accuracy better than 2 nm. The cells spectra were acquired under both specular reflectance and transmittance, using a tungsten halogen light source with a Nikon NCB11 filter, and a 20 × 0.50 NA objective. Reflectance spectra were normalized using a standard silver mirror (Thorlabs Inc.). All spectra were smoothed with a running average of three data points and plotted using Matlab (2019a).

#### TEM

Fish were euthanized in MS222 (E10521, Sigma-Aldrich) then relevant skin portions were dissected and placed in PBS (P3813, Sigma-Aldrich) solution at room temperature. Tissue was mechanically dissected using a scalpel and then sonicated for 10 min. The obtained suspension was centrifuged for 5 min at 4000 RPM, after which the pellet was discarded, and the supernatant was kept and vortexed for 1 min. This process was repeated twice. A total of 10 µl drop of obtained suspension was placed onto a copper slot TEM grid coated with Formvar/carbon and was allowed to settle for 5 min. After which the grid was carefully blotted dry with help of a piece of filter paper. The TEM grids were imaged in a Tecnai Spirit electron microscope (FEI, Hillsboro, OR) operating at 80 kV equipped with an Ultrascan 4000 digital camera (Gatan Inc, CA).

#### Raman microspectroscopy

Samples were prepared by sandwiching prepared tissues or crystals between quartz coverslips (Electron Microscopy Sciences, Cat. No. 72255-02) and glass microscope slides (VWR, Cat. No. 16004-422). All data were collected at RT using a home-built Raman microscope^56^. We used a 514-nm line of an argon-ion laser (CVI Melles Griot, 35-MAP-431-200), which was passed through a clean-up filter (Semrock, LL01-514-25) and then directed into a modified inverted microscope (Olympus IX71). Excitation light (~30 mW at the sample) was directed to the sample using a dichroic mirror (Semrock, LPD01-514RU-25×36-1.1) and a 60× water-immersion objective (Olympus, UPLSAPO60XW). Spontaneous Raman Stokes scattering was collected through the same objective, filtered (Semrock, LP02-514RE-25) to remove any residual excitation light or Rayleigh scattering, and then directed into a 320-mm focal length (*f*/4.1 aperture) imaging spectrometer (Horiba Scientific, iHR 320) through a 400 µm pinhole (Thorlabs) and a 50-μm slit, and dispersed using a 1200 g/mm grating. Individual spectra were collected for 10–15 s acquisitions (×10–15) from 500–3700 cm^−1^ with high gain enabled on a liquid nitrogen cooled, back illuminated deep-depletion CCD array (Horiba Scientific, Symphony II, 1024 × 256 px, 26.6 mm × 6.6 mm, 1 MHz repetition rate). Bright field images were collected using a USB 2.0 camera (iDS, UI-1220-C). Daily calibration of imaging spectrometer was done using neat cyclohexane (20 μl in a sealed capillary tube). Bandpass and accuracy were found to be <12 and ± 1 cm^–1^, respectively. All Raman spectra were corrected by applying a baseline polynomial fit (Lab Spec 6 software).

#### Isolation of cells for scRNA-sequencing

Fish were euthanized in MS222(E10521, Sigma-Aldrich) and the stripe and interstiped regions were microdissected from fish expressing both *pnp4a*:mem-mCherry and *pnp4a*:nnucEos. Stripe and interstipe regions were enzymatically dissociated separately with Liberase (0.25 mg/ml in dPBS, LIBDL-RO, Roche) at 25 °C for 15 min followed by manual trituration with increasingly narrower flame polished glass pipette for 3 min at a time for three times. Cells suspensions were then filtered through a 70 μm nylon cell strainer to obtain a single-cell suspension. Liberated cells were resuspended in 1% BSA (A2153, Sigma-Aldrich)/5% FBS (F2442, Sigma-Aldrich) in dPBS before FACS purification. This was done for samples collected from four different fish that were processed in two different cycles, combining skin samples from two different fish for each cycle. The flow cytometry experiments were performed on a BD FACSAria II SORP sorter (BD Biosciences, San Jose, CA, USA) coupled with BD FACSDiva software (BD Biosciences) for instrument operation, data acquisition, and analysis. A 637 nm and a 488 nm laser were utilized for fluorophore excitation and a 100-μm nozzle was used to generate single droplets under 20PSI sheath pressure. The applied settings were as follows: forward light scatter (FSC) detector photomultiplier tube (PMT) gain setting = 80 V with a 1.5 neutral density filter; side light scatter (SSC) detector PMT gain setting = 90 V; FSC threshold = 10,000; PE-Texas Red (PE TX Red) channel PMT gain setting = 280 V; fluorescein isothiocyanate channel PMT gain setting = 280 V (an exemplification the gating strategy is provided in Supplementary Fig. 20). Sample dilution and flow rate were adjusted to optimal event recordings for 96-well plate single cell sorts (below 500 processed events per sec). The population of zebrafish skin iridophores was designated based on their FSC and SSC characteristics and back-gating on fluorescence. Control zebrafish skin samples were used to gate out the highly auto fluorescent cells among the members of this population. Fluorescently labeled from either the stripe or interstripe skin samples were sorted separately. Single cells with high mCherry and Eos expression were collected into 3 μL of smart-scrub lysis buffer (0.2% Triton X-100 (Sigma), 0.1 U/μl RNAse inhibitor (NEB)) using “single cell” indexed sorting mode. Five indexed 96-well plates were collected for each skin band. After sorting, the samples were spun down at 3166 r.c.f. at 4 °C for 2 min in an Eppendorf tabletop 5810 R centrifuge (Eppendorf AG, Hamburg, Germany) and stored at −80 °C until further processing.

#### scRNA-seq library preparation

Four plates of each skin band were selected for sequencing. cDNA was prepared from sorted cells as described previously by Cembrowski et al.^57^ with minor modifications. Reverse transcription, PCR, purification, tagmentation, and library quantification were performed as described. Libraries from eight plates were pooled equimolar and were sequenced on a NextSeq 550 high-output flowcell with 25 bases in read 1 to read the 1 bp spacer, 8 bp barcode, 10 bp UMI as described in Cembrowski et al., 8 bases in the i7 index read, and 50 bases in read 2 (cDNA). phiX control library (Illumina) was spiked in at a final concentration of 15% to improve color balance in read 1. Libraries were sequenced to an average depth of 39,875,610 ± 8,449,306 reads (mean ± standard deviation (SD)).

#### scRNA-seq analysis

Sequencing adapters were trimmed from the reads with Cutadapt v2.10 (Martin, 2011) prior to alignment with STAR v2.7.5a (Dobin et al., 2013) to the *D. rerio* GRCz11.94 genome assembly from Ensembl (ensembl.org). Gene counts were generated with the STARsolo algorithm using the following additional parameters: “–soloType CB_UMI_Simple–soloCBwhitelist smartscrb_whitelist.txt–soloCBstart 2–soloCBlen 8–soloUMIstart 10–soloUMIlen 10 -soloBarcodeReadLength 0–soloCBmatchWLtype 1MM_multi_pseudocounts–soloStrand Forward–soloFeatures Gene–soloUMIdedup 1MM_All–soloUMIfiltering MultiGeneUMI–soloCellFilter None”. The full set of 384 barcodes designed for this assay was used as the whitelist (Supplemental file “Q.index.txt”), and the full description of Cutadapt and STAR parameters is provided in Supplemental file “pipeline_parameters.txt”. Gene counts for the subset of barcodes used in each library were extracted using custom R scripts.

Raw counts representing the enumerated expression for 32,618 features for 768 cells was read into R (v3.6.1) in table form with features in rows and cells in columns. This matrix was then converted into a single-cell experiment object using the “SingleCellExperiment” function supported in the “SingleCellExperiment” library (v1.8.0). Features observed to not have a count greater than zero for at least one cell were removed. Calculation of quality control metrics and screening for low quality cells was accomplished using a combination of functions supported in the “scater” library (v1.14.6). Specifically, the “perCellQCMetrics” function was used to calculate metrics per cell, the “perFeatureQCMetrics” function used to calculate metrics per feature, and the “quickPerCellQC” function used to identify cells as low quality. In addition, the “plotColData” function was used to visually inspect the number of detected features per cell by the total number of counts to define a range per number of features (>200, <1750) and counts (>0.45e5, <1.45e5) a cell must satisfy to not be considered low quality. The union set of cells identified as low quality were then removed and counts for surviving cells, minus features representing mitochondria genes or spike-in controls, used to create a new cell data object via the “CreateSeuratObject” function supported in the “Seurat” library (v3.1.4). This object was then used in combination with functions supported in the “Seurat” library, under default settings, to generate clusters of cells. Specifically, counts were first normalized using the “SCTransform” function then principal component analysis performed on the normalized counts using the “RunPCA” function. After, the “ElbowPlot” function was used to inspect and define the number of components to pass to the “FindNeighbors” function (*n* = 10). Lastly, the “FindClusters” function was used to generate clusters of cells (resolution = 0.8) that were ultimately visualized and inspected using the “RunUMAP”, “DimPlot”, and “FeaturePlot” functions. To test for dysregulated features between clusters, the “FindMarkers” function was used. Trajectory analysis was also performed on the same counts for surviving cells and features using a combination of function calls under default settings that are supported in the “M3Drop” library (v1.12.0) and “monocle” library (v2.14.0). Specifically, the “M3DropFeatureSelection” function was used to find differentially expressed features then counts for those features used to construct a new cell data object using the function called “newCellDataSet”. This data object was then passed to the following functions in sequence to produce and ultimately visualize the trajectory: “setOrderingFilter”, “estimateSizeFactors”, “reduceDimension”, “”orderCells”, and “plot_cell_trajectory”. The data discussed in this publication have been deposited in NCBI’s Gene Expression Omnibus (Edgar et al., 2002) and are accessible through GEO Series accession number GSE144734 (https://www.ncbi.nlm.nih.gov/geo/query/acc.cgi?acc=GSE144734).

#### Cryo-scanning electron microscopy

Fish were euthanized in MS222 (E10521, Sigma-Aldrich) then relevant skin portions were sectioned and placed in PBS (P3813, Sigma-Aldrich) solution at room temperature. The sections were then sandwiched between two metal discs (3 mm diameter, 0.1 mm cavities) and cryo-immobilized in a high-pressure freezing device (HPM10; Bal-Tec). The frozen samples were mounted on a holder under liquid nitrogen and transferred to a freeze-fracture device (BAF60; Bal-Tec), using a vacuum cryo-transfer device (VCT 100; Bal-Tec), where they were coated with a 3-nm-thick layer of Pt/C. Samples were then observed by high-resolution SEM (Ultra 55, Zeiss) using secondary electron/backscattered electron and an in-lens detector, maintaining the frozen hydrated state by using a cryo-stage operating at a working temperature of −120 °C. Measurements of crystal thickness and cytoplasm spacing were taken from the cryo-SEM micrographs.

#### Malachite green staining

Fish were euthanized in MS222 (E10521, Sigma-Aldrich) then relevant skin portions were sectioned and placed in a PBS (P3813, Sigma-Aldrich) solution containing 25–50 μM malachite green (38800, Sigma-Aldrich), at room temperature for 30 min before imaging.

#### Reflectivity simulations

Reflectivity was simulated based on metamorphic data obtained from cryo-SEM (crystal thicknesses, spacing, orientation, and number of layers), using a Monte Carlo transfer matrix calculation^58^. The reflectivity was simulated by averaging 500 runs, assuming either normal incident, or in the angle depends studies between 0° and 70°. Each one of the layers was characterized by two variables: *n*_*j*_, a refractive index, and *d*_*j*_, which is the layer thickness randomly picked from the experimental distribution. Thus, for each layer we defined the following 2 × 2 matrix:

1 $$\left( {\begin{array}{*{20}{c}} {{{{\mathrm{cos}}}}{{\beta }}_{{j}}} & { - \frac{{{i}}}{{{{n}}_{{j}}}}\sin {{\beta }}_{{j}}} \\ { - {{in}}_{{j}}{{{\mathrm{sin}}}}{{\beta }}_{{j}}} & {\cos {{\beta }}_{{j}}} \end{array}} \right)\,{{{\mathrm{where}}}}\,\beta _j = \frac{{2\pi }}{\lambda }n_jd_j.$$

The set of *k* double layers was characterized by the following reflectivity 2 × 2 matrix:

2 $$M_j = \mathop {\prod }\limits_{j = 1}^{j = 2k} mj.$$

Reflectivity was obtained using the following equation:

3 $$R = \left| {\frac{{\left( {m_{11} + m_{12}} \right) - \left( {m_{21} + m_{22}} \right)}}{{\left( {m_{11} + m_{12}} \right) + \left( {m_{21} + m_{22}} \right)}}} \right|.$$

The refractive index for the guanine crystals was set as 1.83. We neglected the weak dependence of the refractive index on wavelength and assumed that the interfaces were parallel.

#### Figures plotting and assembly

Graphs were plotted using MATLAB (2019a) or Prism (v8), and figures were assembled using Illustrator (v2020) and photoshop (cc2019).

### Statistics and reproducibility

Sample size was based on previous experience. No statistical method was used to predetermine sample size. Unless otherwise noted, each experiment was repeated three or more times. Data shown in column graphs represent mean ± standard error of the mean (SEM) or mean ± SD, as indicated in the figure legends, and individual data points are plotted. Statistical analysis was performed with GraphPad Prism 6.0. Details of statistical testing can be found in the figure legends and in the Source data file. All datasets were tested for Gaussian distribution using the Kolmogorov–Smirnov test.

### Reporting summary

Further information on research design is available in the Nature Research Reporting Summary linked to this article.

## Supplementary information

Supplementary Information

Supplementary Table 1

Supplementary Table 2

Supplementary Movie S1

Supplementary Movie S2

Supplementary Movie S3

Supplementary Movie S4

Supplementary Movie S5

Supplementary Movie S6

Supplementary Movie S7

Supplementary Movie S8

Description of Additional Supplementary Files

Reporting Summary

## Data Availability

Data supporting the findings of this manuscript are available from the corresponding authors upon reasonable request. A reporting summary for this article is available as a Supplementary Information file. All sequencing data that support the findings of this study have been deposited in the National Center for Biotechnology Information Gene Expression Omnibus (GEO) and are accessible through the GEO Series accession number GSE144734. Source data are provided with this paper.

## Source data

Source Data
