# Author Correction: In situ differentiation of iridophore crystallotypes underlies zebrafish stripe patterning

**DOI:** 10.1038/s41467-022-32152-z

**Published:** 2022-07-26

**Authors:** Dvir Gur, Emily J. Bain, Kory R. Johnson, Andy J. Aman, H. Amalia Pasolli, Jessica D. Flynn, Michael C. Allen, Dimitri D. Deheyn, Jennifer C. Lee, Jennifer Lippincott-Schwartz, David M. Parichy

**Affiliations:** 1 HHMI Janelia Research Campus, Ashburn, VA USA; 2 National Institute of Child Health and Human Development, NIH, Bethesda, MD USA; 3 Department of Biology, University of Virginia, Charlottesville, VA USA; 4 Department of Biology and Department of Cell Biology, University of Virginia, Charlottesville, VA USA; 5 Bioinformatics Section, National Institute of Neurological Disorder and Stroke, NIH, Bethesda, MD USA; 6 National Heart, Lung, and Blood Institute, NIH, Bethesda, MD USA; 7 Marine Biology Research Division, Scripps Institution of Oceanography, University of California, San Diego, La Jolla, CA USA

**Keywords:** Biophysics, Pattern formation

Correction to: *Nature Communications* 10.1038/s41467-020-20088-1, Article published online 15 December 2020

In this article the author name H. Amalia Pasolli was incorrectly written as H. Amalia Pasoili. The original article has been corrected.

