# Supplementary material for: In situ differentiation of iridophore crystallotypes underlies zebrafish stripe patterning
Source: Nat Commun. 2020 Dec 15;11:6391. doi: 10.1038/s41467-020-20088-1 (PMC7738553; doi:10.1038/s41467-020-20088-1)
Supplement: Supplementary file 1 — Supplementary Information [file 41467_2020_20088_MOESM1_ESM.pdf]

***In situ* differentiation of iridophore crystallotypes underlies zebrafish stripe patterning**

Dvir Gur[#,1,2], Emily Bain[#,3], Kory Johnson[4], Andy J. Aman[3], Amalia Pasoili[1], Jessica D. Flynn[5], Michael C. Allen[6], Dimitri D. Deheyn[6], Jennifer C. Lee[5], Jennifer Lippincott-Schwartz[*,1], David Parichy[*,3]

Supporting Information contains:

- **Supplementary Data Figures**
- **References**

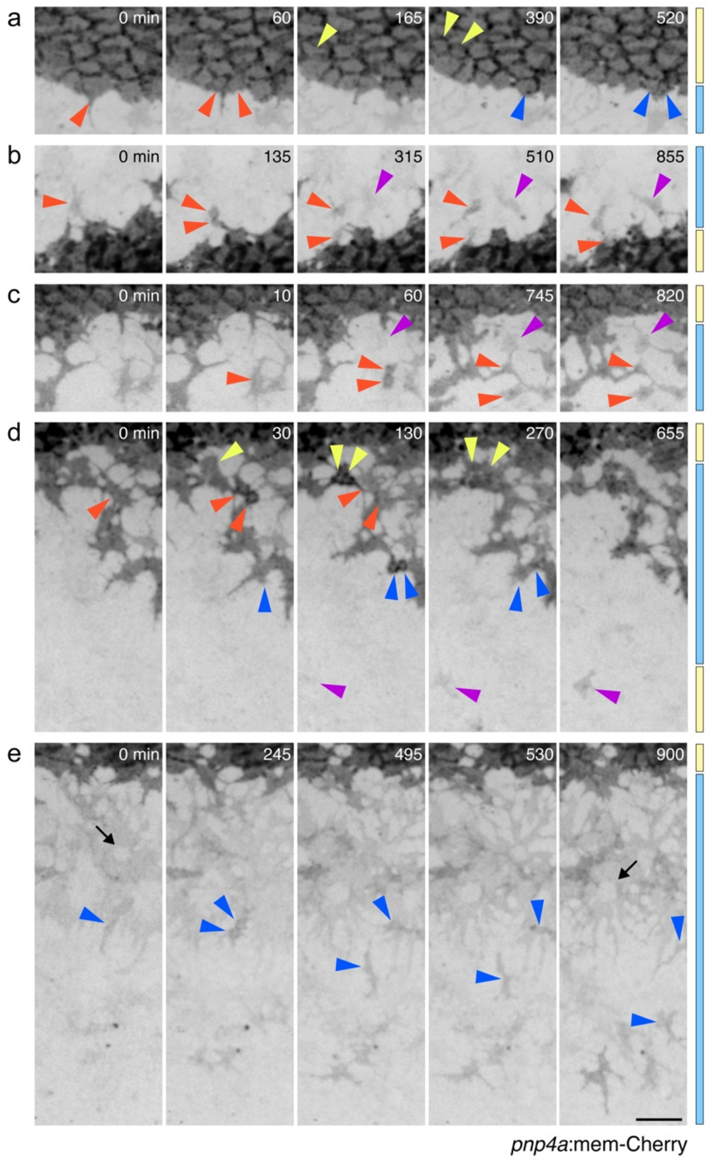

Supplementary Figure 1. Live imaging reveals proliferation, differentiation and migration but not dynamic changes in state. (a) *pnp4a*:mem-Cherry (inverted pixel values) reveals dividing cells at interstripe edge (orange and blue arrowheads) and within interstripe. Bars at right indicate approximate boundary between interstripe (yellow) and stripe (blue) regions and numbers in frames indicate sequential day of imaging. **(b)** Division of a cell newly expressing *pnp4a* within the prospective stripe (orange) with onset of *pnp4a* expression by an adacent cell (purple). **(c)** Division (orange) and appearance (purple) of *pnp4a*+ cells within the developing ventral primary stripe. **(d)** Division of *pnp4*+ cells (orange, yellow, blue) within stripe and appearance of new *pnp4a*+ cell (purple) at site likely corresponding to prospective secondary ventral interstripe. **(e)** Division and migration of *pnp4*+ cells within ventral stripe (blue, purple). Black arrows indicate cells having morphologies and minimal motility typical of xanthophores, which also express low levels of *pnp4a* (Saunders et al., 2019). Stages of larvae shown 7.5–8.0 SSL (1). Examples shown are representative of 10 larvae examined at these stages. Scale bar in e for a–e, 40 μm.

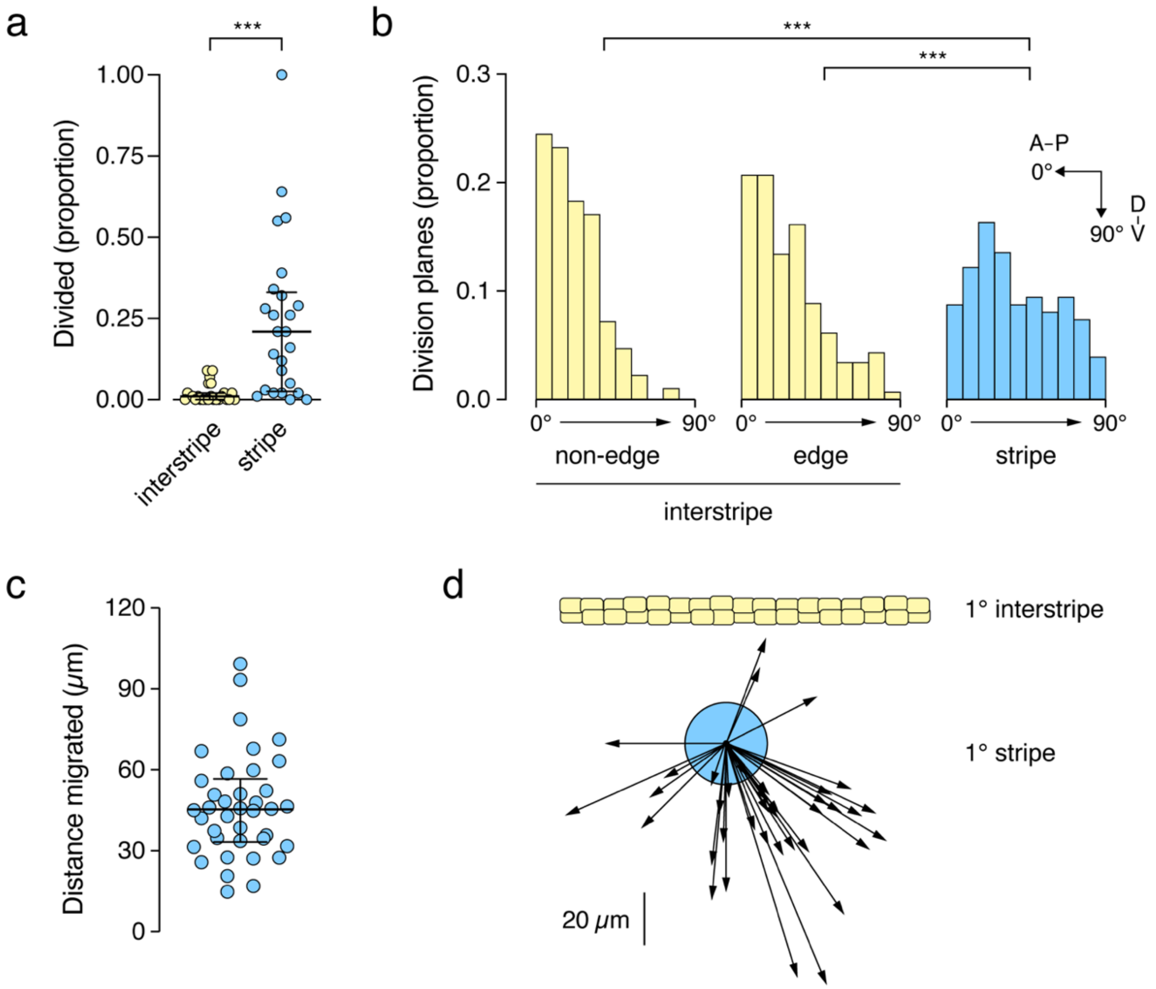

Supplementary Figure 2. Division and migration of *pnp4a*+ cells in time-lapse imaging. **(a)** Loose iridophores of stripes were more likely to divide than dense iridophores of interstripes (median ± interquartile range, N=25 larvae with 11,580 total *pnp4*+ cells; χ^2=633, P<0.0001, d.f.=1; Wilcoxon test comparison of proportions, Z=4.6, P<0.0001 two sided, ***). **(b)** Proportions of cells having planes of division ranging from anteroposterior (0°) to dorsovental (90°) for populations within the interstripe and bounded only by other cells of the interstripe (non-edge), in the interstripe but not completely bounded by other cells of the interstripe (edge), or within the stripe. Division plane variances differed significantly among iridophores at different positions (Levene's test, $F_{2,333}$=12.7, P<0.0001), as expected for the more uniform planes of division exhibited by stripe as compared non-edge and edge iridophores. Paired comparisons of means likewise indicated significantly greater values for division planes of stripe iridophores than either non-edge or edge iridophores (Wilcoxon Z=5.4 and 3.9, respectively, both P<0.0001; ***). **(c)** Distances migrated (median ± interquartile range) by loose *pnp4*+ cells of the prospective ventral primary stripe. Only cells moving greater than one half the diameter of a typical loose iridophore (~31 μm) were included (N = 38 cells total from 4 different larvae at 7.0–7.5 SSL when the ventral primary stripe is first developing). **(d)** Directions and distances migrated by same cells illustrated in c. A majority of cells moved ventrally and posteriorly from their origin. Blue circle indicates average diameter of loose iridophores.

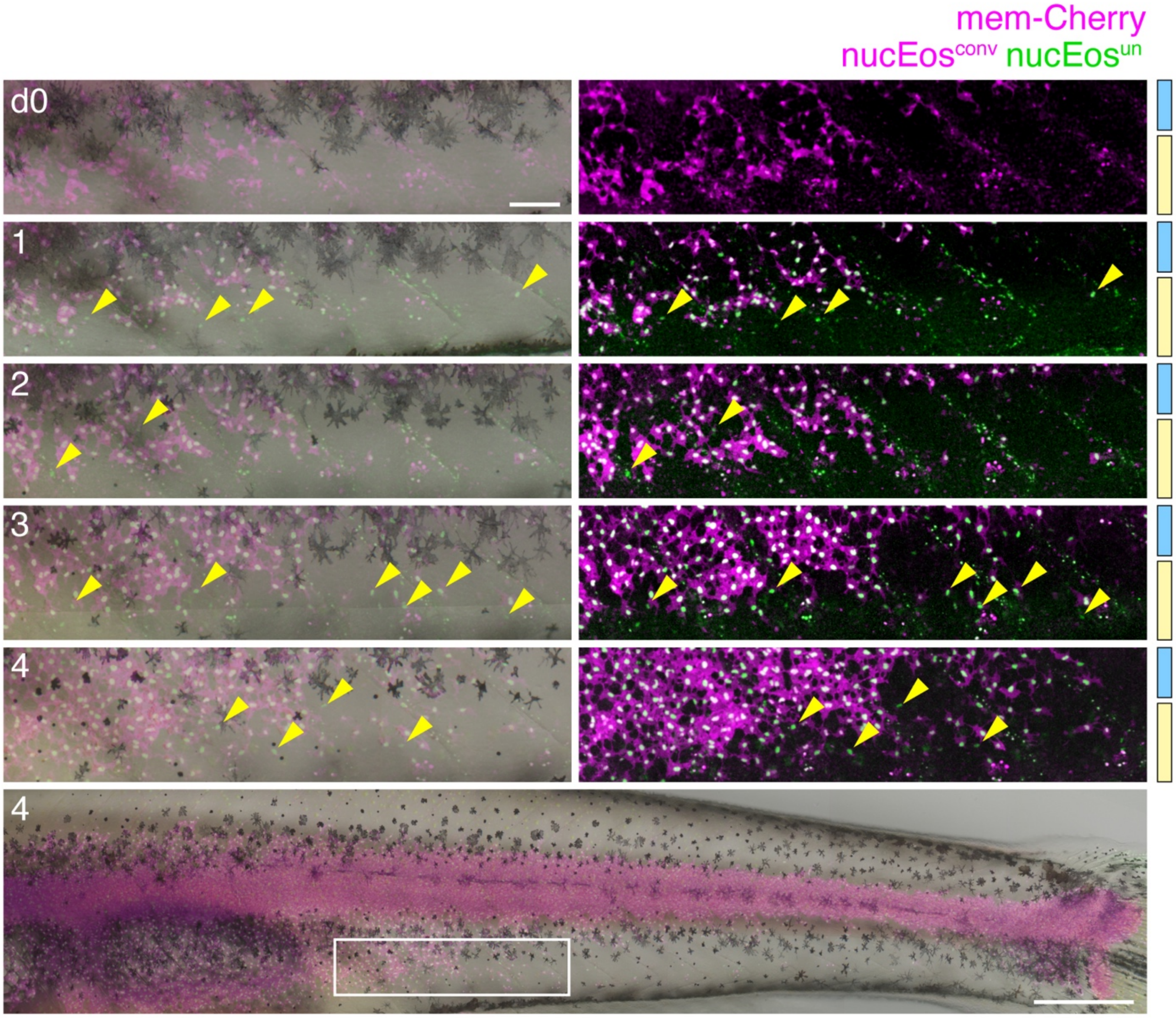

Supplementary Figure 3. Differentiation of iridophores contibuting to secondary interstripe. To identify newly appearing cells, whole fish were exposed to UV light daily to photoconvert all *pnp4*:nucEos from green (nucEos^{un}) to red (nucEos^{conv}; here displayed in magenta). On the following day, cells exhibiting nucEos^{un} but not nucEos^{conv} have arisen. Older cells typically exhibit both nucEos^{un} and nucEos^{conv} (white) and begin to exhibit the weaker fluorophore *pnp4*:mem-Cherry. At d0, immediately after photoconversion, all *pnp4*+ nuclei are magenta. At d1, green nuclei exhibiting only nucEos^{un} are evident (e.g., arrowheads). After subsequent rounds of photoconversion, nuclei newly expressing nucEos^{un} continued to appear (d2–4). Bottom panel at low magnification illustrates region highlighted above. Images of this individual are representative of 8 total individuals examined. Scale bars, 100 µm (d0, for details d0–d4), 500 µm (d4, for overview).

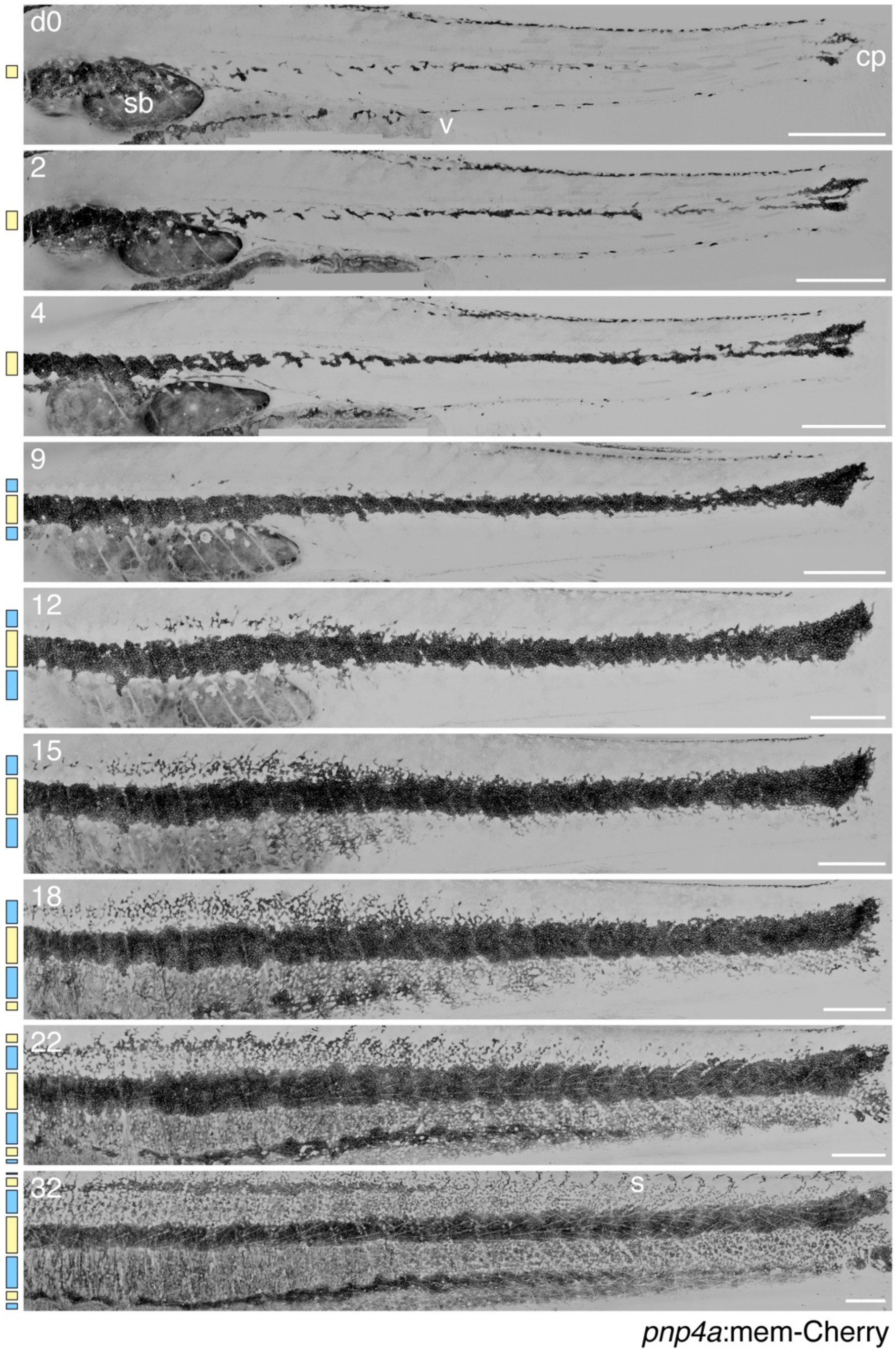

Supplementary Figure 4. Iridophore pattern development across anteroposterior axial levels. Shown is a representative individual (of 10 total individuals) imaged daily for 28 d (6.0–11.0 SSL) with images rescaled to control for growth. Bars at left represent progressive appearance of *pnp4*:mem-mCherry+ cells contributing to interstripes (yellow) and stripes (blue). sb, posterior lobe of swimbladder; v, vent; cp, caudal peduncle; s, scales (d28). Images are rescaled for growth. Scale bars, 500 μm.

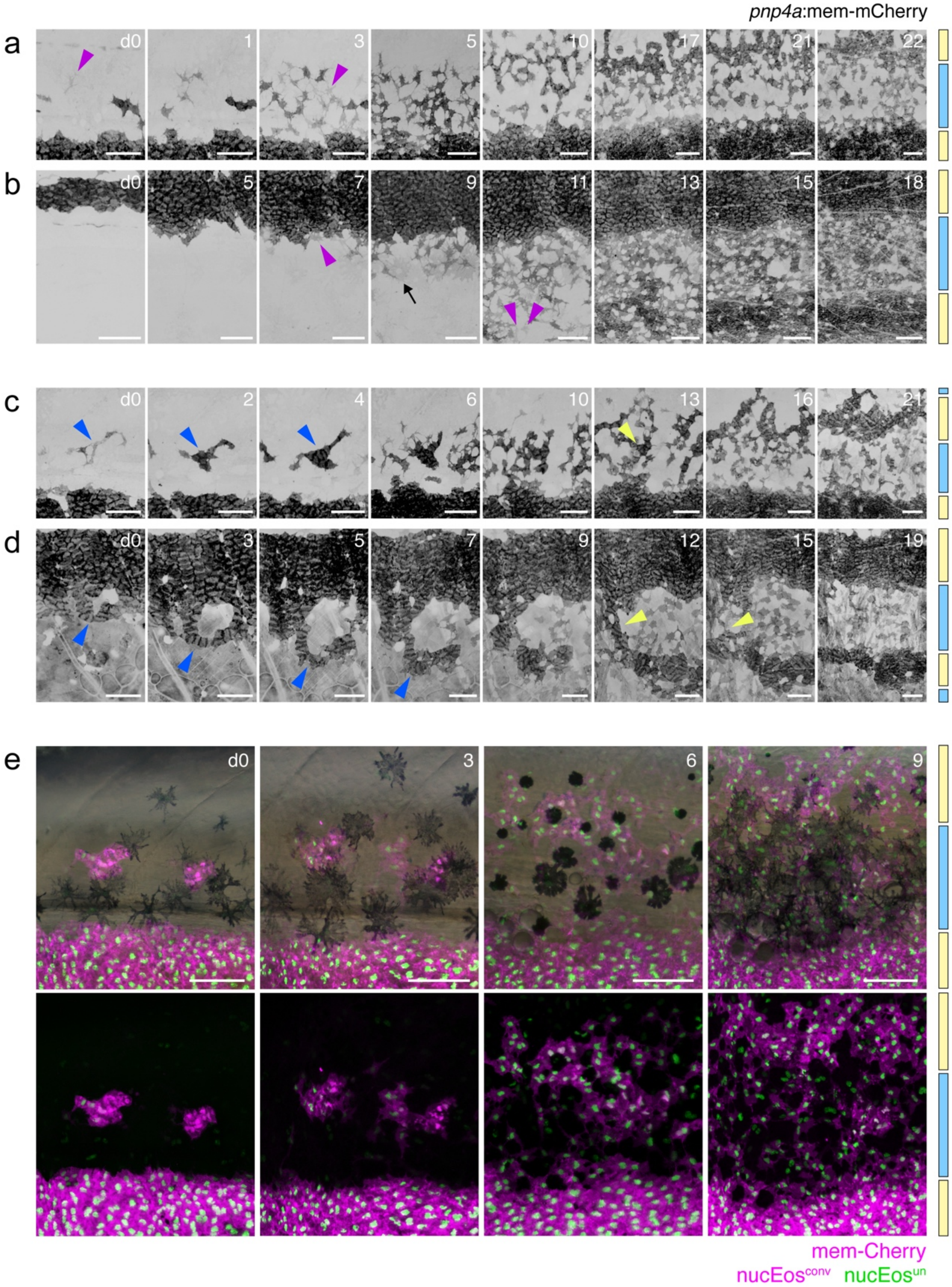

pnp4a:mem-mCherry
a
d0
1
3
5
10
17
21
22
b
d0
5
7
9
11
13
15
18
c
d0
2
4
6
10
13
16
21
d
d0
3
5
7
9
12
15
19
e
d0
3
6
9
mem-Cherry
nucEosconv nucEosun

Supplementary Figure 5. Repeated imaging of iridophore pattern formation and nucEos fate mapping of iridophore clusters within the dorsal stripe. (a–d) Details from stitched images that covered the anterior–posterior and dorsal–ventral boundaries of pattern formation on the body, examples of which are provided in Supplementary Movies S6 and S7. Bars at right indicate prospective interstripe (yellow) and stripe (blue) regions. **(a,b)** Appearance of *pnp4a*+ cells (purple arrowheads) within the dorsal stripe (a) and ventral stripe (b), and emergence of the secondary interstripes. Black arrow indicates cell with typical xanthophore morphology, weakly expressing *pnp4a*:mem-Cherry. Images are representative of most or all regions in 10 fish examined. **(c,d)** Examples of patterning observed in a minority of wild-type fish (2 of 10 examined). In (c), several iridophores arise in an initially dense arrangmenet (blue arrowheads) within the prospective dorsal stripe and subsequently assumes a more dispersed arrangement (yellow arrowhead) within the stripe. In (d), a ventrally extending group of densely packed iridophores (blue arrowheads) ultimately becomes separated from the primary interstripe (yellow arrowheads) and contributes to the second ventral interstripe. **(e)** Photoconversion of *pnp4a*:nucEos expressed by iridophores in small clusters within the developing dorsal stripe revealed cells retaining photoconverted fluorophore (white nuclei) within the dorsal interstripe (left cluster) and within the dorsal stripe (right cluster). Some additional cells within the marked regions began expressing *pnp4a*:nucEos only after photoconversion (green nuclei). Images shown are representative of photoconversion results observed for small clusters of converted cells in 3 different larvae. Fish across all panels range from stages 7.0–10.5 SSL with images rescaled to control for growth. Scale bars, 100 μm.

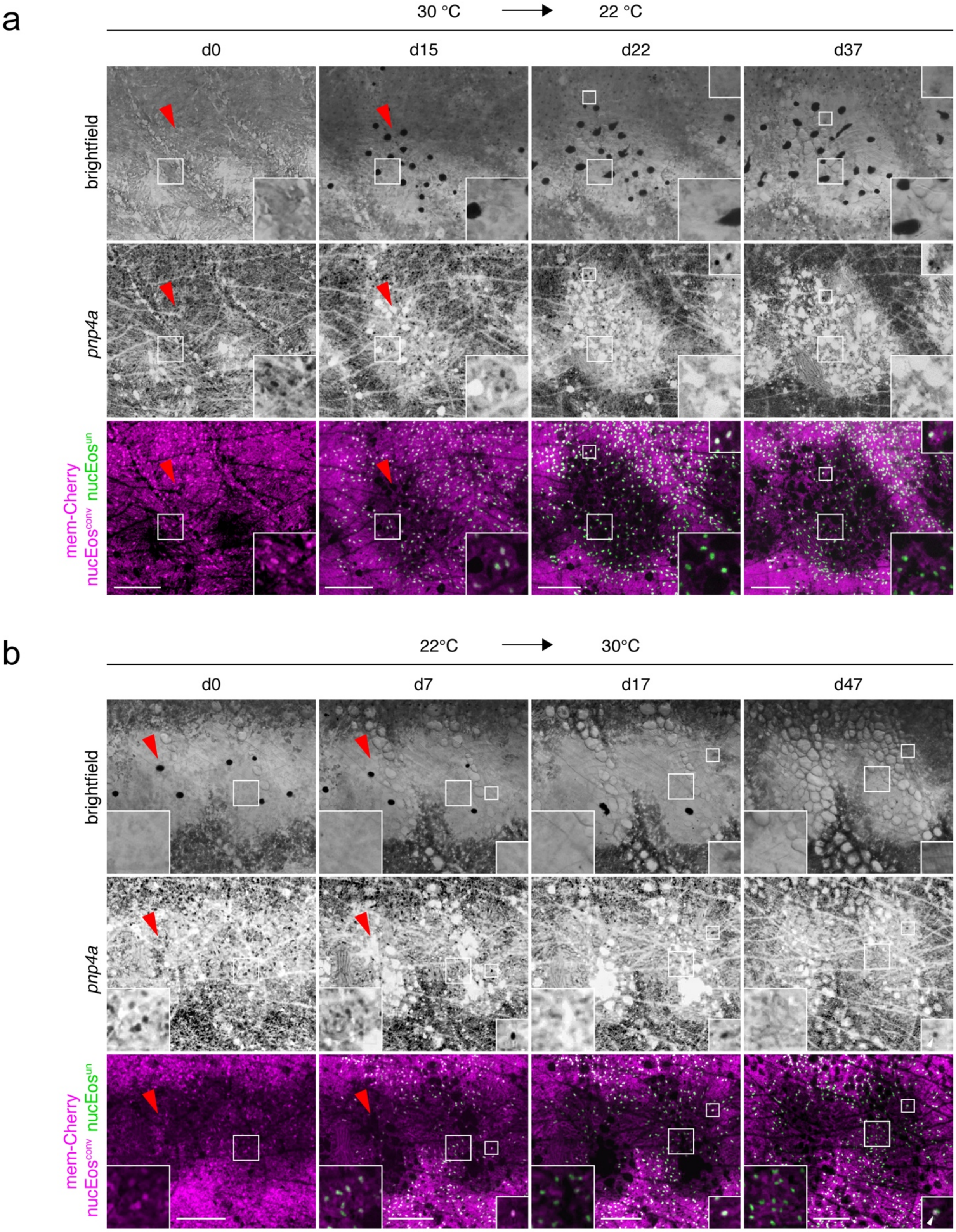

a
30 °C
22 °C
d0
d15
d22
d37
brightfield
pnp4a
mem-Cherry
nucEos^conv
nucEos^un
b
22°C
30°C
d0
d7
d17
d47
brightfield
pnp4a
mem-Cherry
nucEos^conv
nucEos^un

Supplementary Figure 6. Differentiation of new iridophores during pattern remodeling. Shown are details of repeatedly imaged, representative adult fish homozygous for the temperature sensitive allele *mitfa*^{vc7}. Fish were stably transgenic to express *pnp4a*:mem-Cherry in all iridophores, marking cell outlines, and were transiently transgenic for *pnp4a*:nucEos, thereby expressing photoconvertible fluorophore mosaically among iridophores. nucEos was photoconverted and fish were then shifted between temperatures to promote melanophore development or death. **(a)** Shift from restrictive temperature (30°C) to permissive temperatures (22 °C) allowed melanophore differentiation by ~d2, continuing through ~d22 (upper panels, brightfield). The appearance of melanophores was followed by a gradual remodeling of iridophore pattern from a relatively dense to a looser arrangement (middle panels, *pnp4a*). Photoconverted nuclei (magenta; e.g., lower panel, inset on d0) gradually acquired unconverted nucEos (white; inset on d15), but many were subsequently lost, accompanied by the appearance of spaces devoid of iridophores (arrowheads). Later, cells expressing only unconverted nucEos increased in abundance (green; e.g., large insets on d22, d47) though other cells retaining photoconverted nucEos remained evident at margins of remodeled regions (small insets on d22, d37). Images shown are representative of observations for total of 6 individual fish examined. **(b)** Reciprocal temperature shift caused the death of melanophores and iridophore pattern remodeling from a loose to denser arrangement. Remodeling proceeded through a period in which regions became devoid of iridophores with photoconverted nucEos (arrowheads). Although some iridophores with photoconverted nucEos persisted through d47 (white; small insets d7–47, and some cells in large inset on d7), iridophores populating regions without melanophores were increasingly likely to exhibit only unconverted nucEos (green; large insets d17, d47), suggesting they were newly differentiating. Images are representative of observations for a total of 4 individual fish examined. Scale bars, 200 μm.

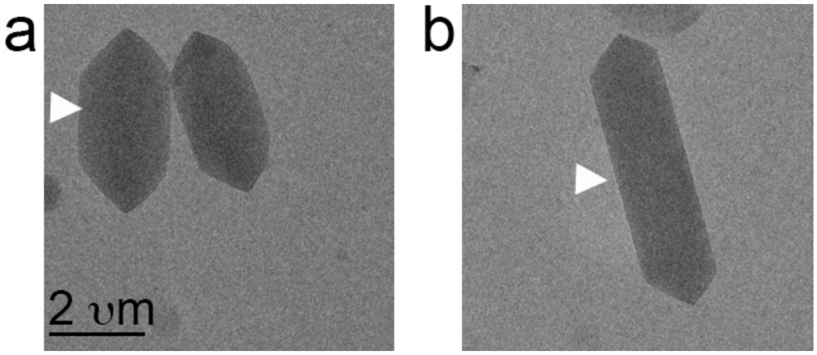

Supplementary Figure 7. TEM micrographs of crystals isolated from stripe iridophores (a) and interstripe iridophores (b), that were used to collect the electron diffraction from figure 3d (left panel) and 3d right panel, respectively. n= 60 crystals for stripe and 57 for interstripe taken from 4 different adult fish.

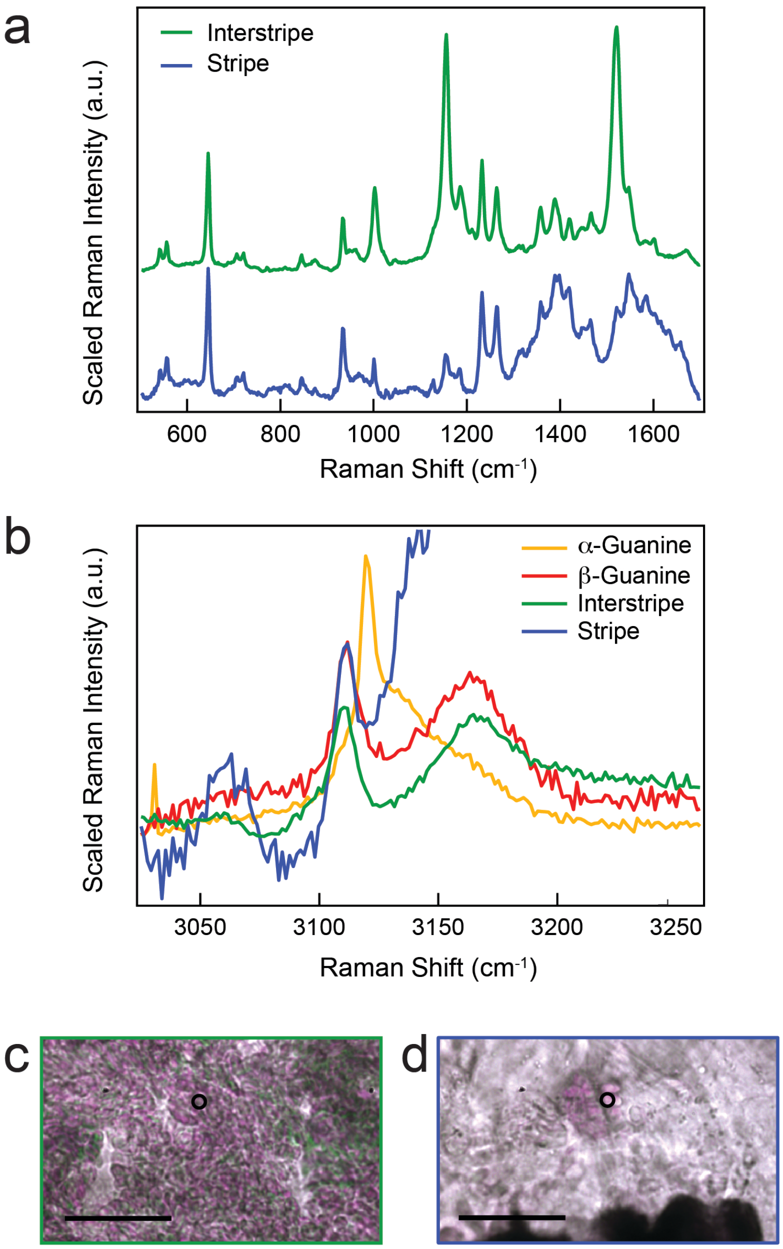

Supplementary Figure 8. Raman micro-spectroscopy of zebrafish stripe and interstripe iridophores. (a) Representative Raman spectra taken from interstripe (green; *n*=9 cells) and stripe (blue; *n*=14) cells in zebrafish tissue taken from 3 different fish. **(b)** High energy peak pattern indicates β-guanine is present in both interstripe and stripe cells. **(c and d)** Brightfield field images of the interstripe (c) and the stripe (d), n = 9 interstripe area (for c) and 14 stripe area (for d) taken from 3 different adult fish. Black circles indicate collection location of Raman spectra in (a). The magenta coloring is due to light diffraction by the crystals and the green coloring is due to absorption from overlying xanthophores. Scale bar, 10 μm.

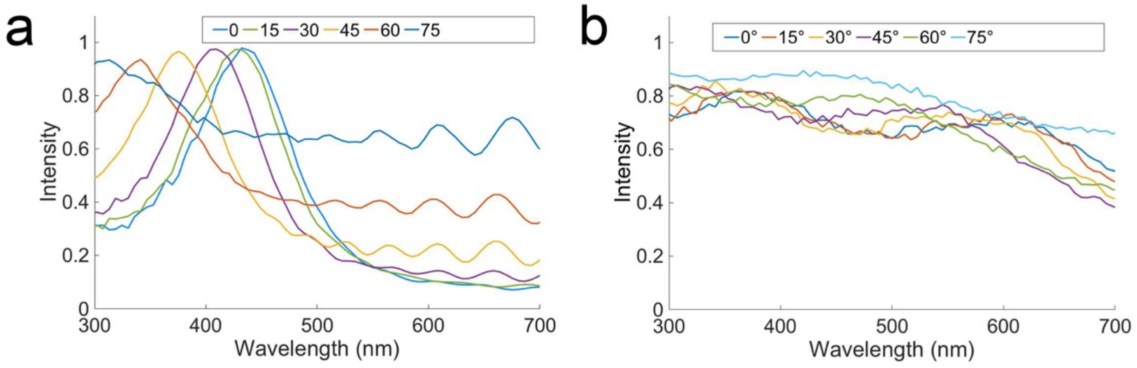

Supplementary Figure 9. Simulations of the expected reflection from iridophores at different angles of incident light. (a,b) Monte Carlo based simulations of the reflection expected from ordered (a) and disordered iridophores (b) at different angles of incident light (0-75°). While there was very little effect over the reflection of the disordered iridophores (b), a clear angular dependence was observed for the ordered iridophores (a) showing a blue shift in the reflected light with increasing angle of incident light.

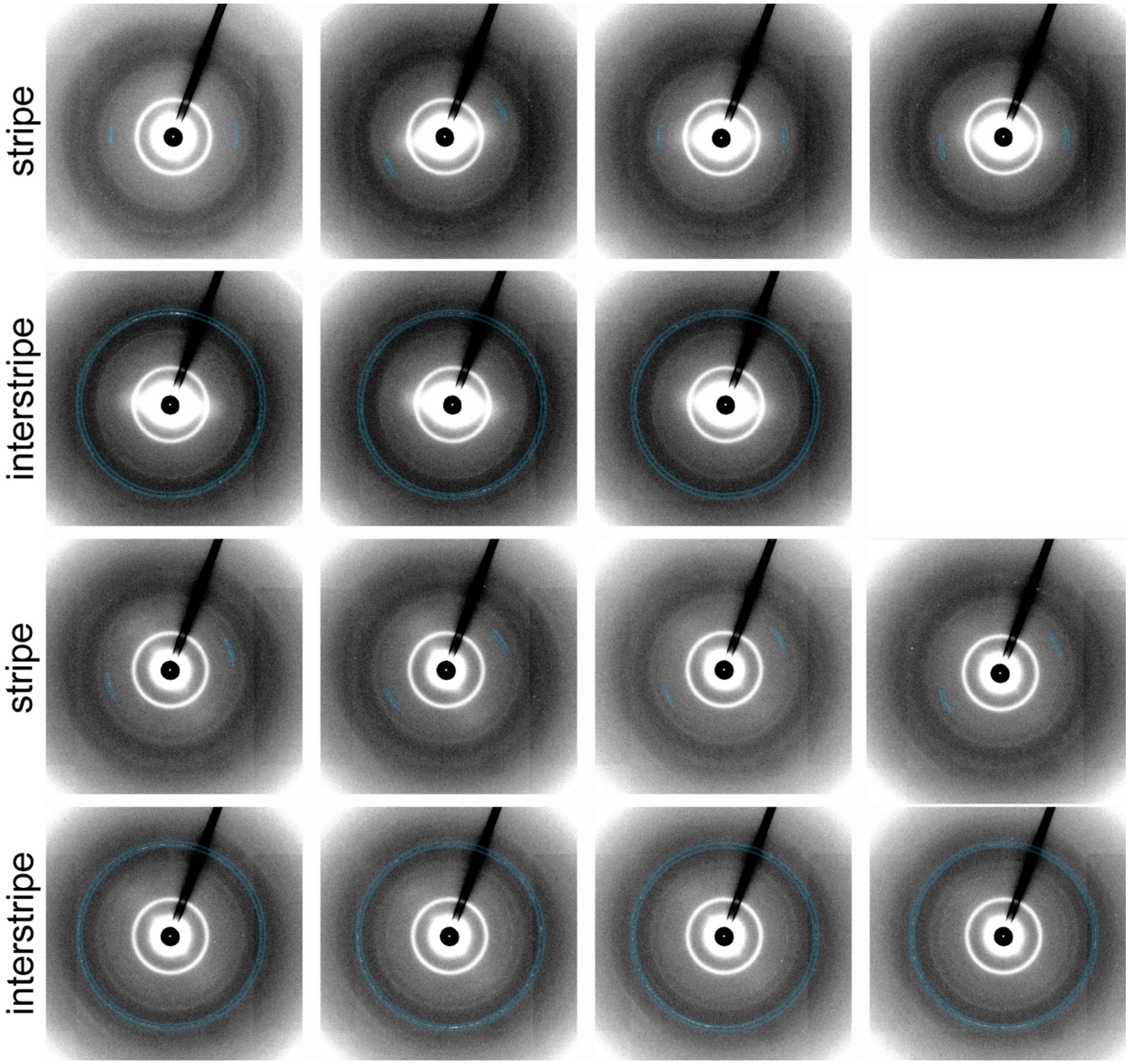

Supplementary Figure 10. Micro X-ray diffractions collected along a line scan of an adult wild-type flank. In this data set of X-ray diffraction taken across the flank of an adult zebrafishlow angular distribution diffractions are found in the diffraction patterns collected along the different stripes regions (stripe), and high angular distribution diffractions are found in the diffraction patterns collected along the different interstripes regions (interstripe).

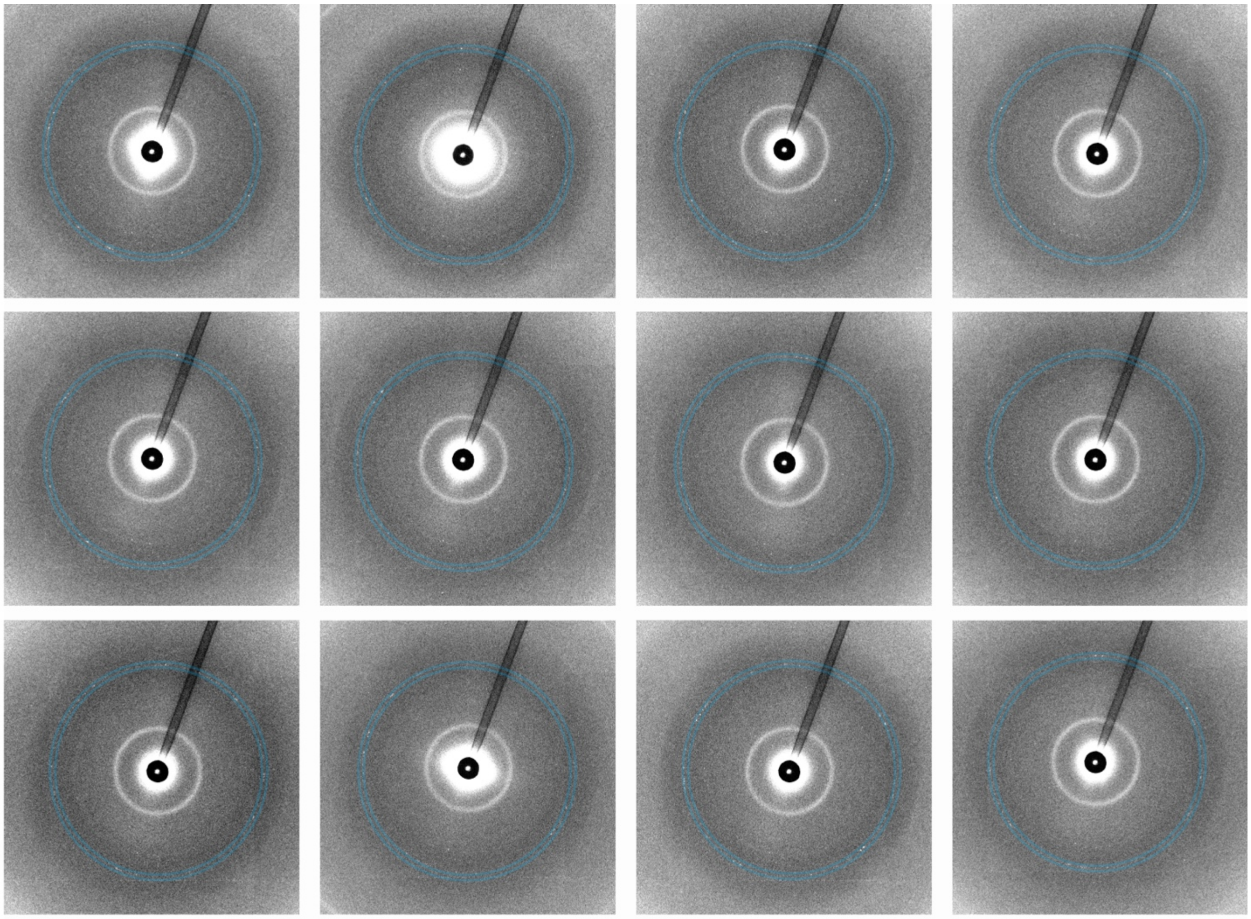

Supplementary Figure 11. Micro X-ray diffractions collected along a line scan of an adult *mitfa*^{w2} mutant flank. In this data set of X-ray diffractions collected along 6 mm line scan across the fish flank, mostly high angular distribution diffractions were found in areas corresponding to both stripe and interstripe in wild-type fish.

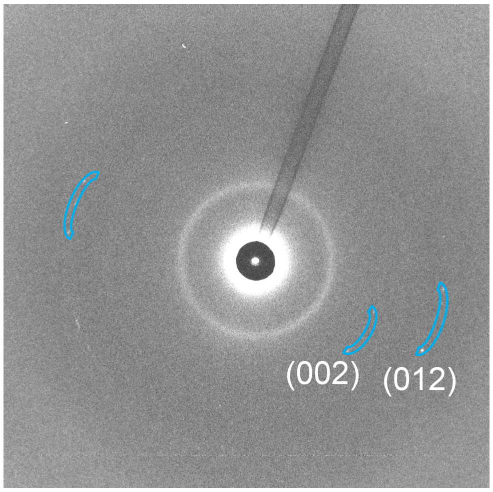

Supplementary Figure 12. Micro X-ray diffraction of an ordered iridophore from the posterior flank of a *mitfa*^{w2} fish. In this X-ray diffraction, low angular distribution diffractions of both the (012) and the (002) planes, typical of the ordered iridophores were observed.

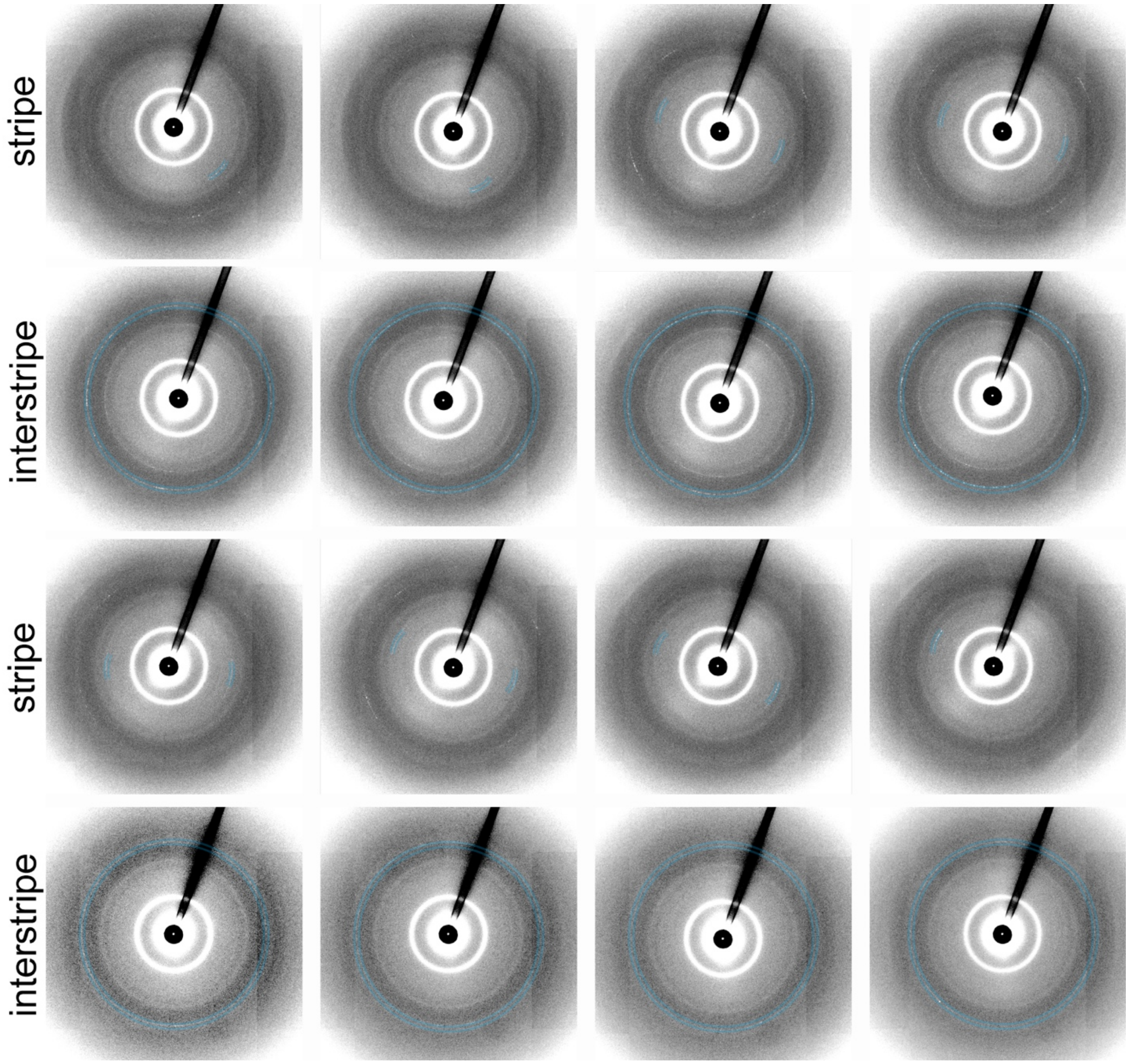

Supplementary Figure 13. Micro X-ray diffractions collected along a line scan of an adult *albino* flank. The overall diffraction pattern resembles that of wild type fish, with highly ordered diffraction patterns of the (002) and (012) diffraction planes throughout the stripe region, and high angular distribution of only the (012) diffraction plane throughout the interstripe region.

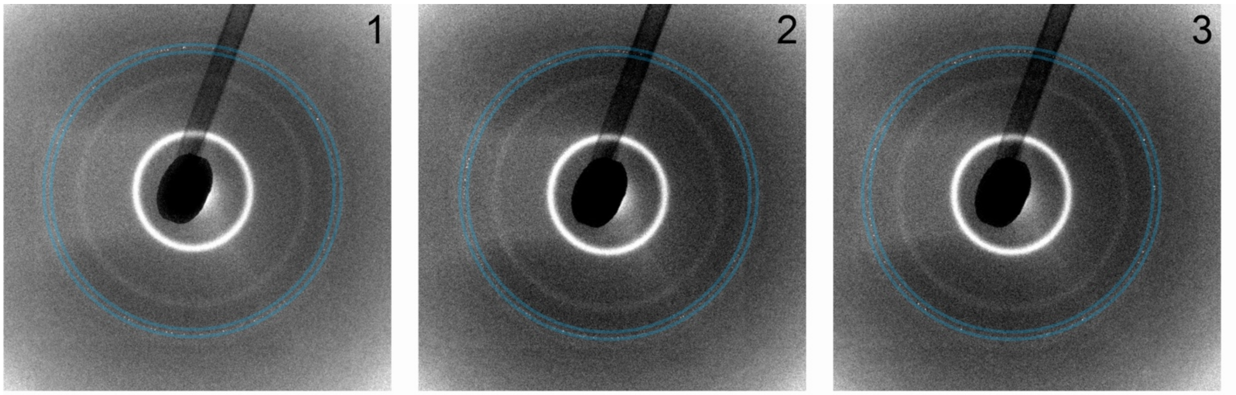

Supplementary Figure 14. Micro X-ray diffraction of a 6.0 SSL larva. X-ray diffraction patterns from a vertical line measured across the trunk of the fish, only high angular distributions of the (012) plane were observed.

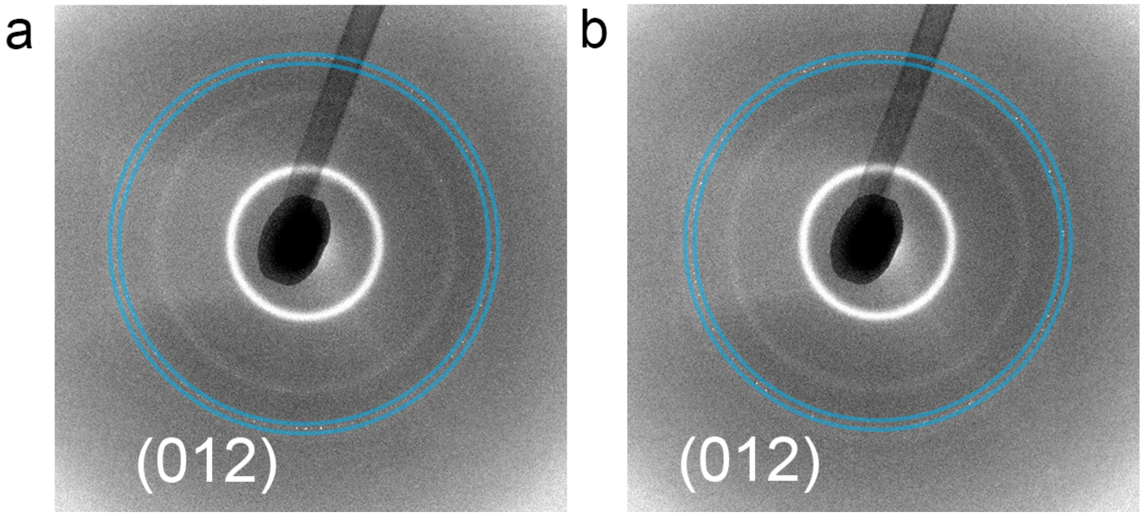

Supplementary Figure 15. Micro X-ray diffraction of a 6.5 SSL larva. (a,b) X-ray diffraction patterns from a vertical line measured across the trunk of the fish, only high angular distributions of the (012) plane were observed.

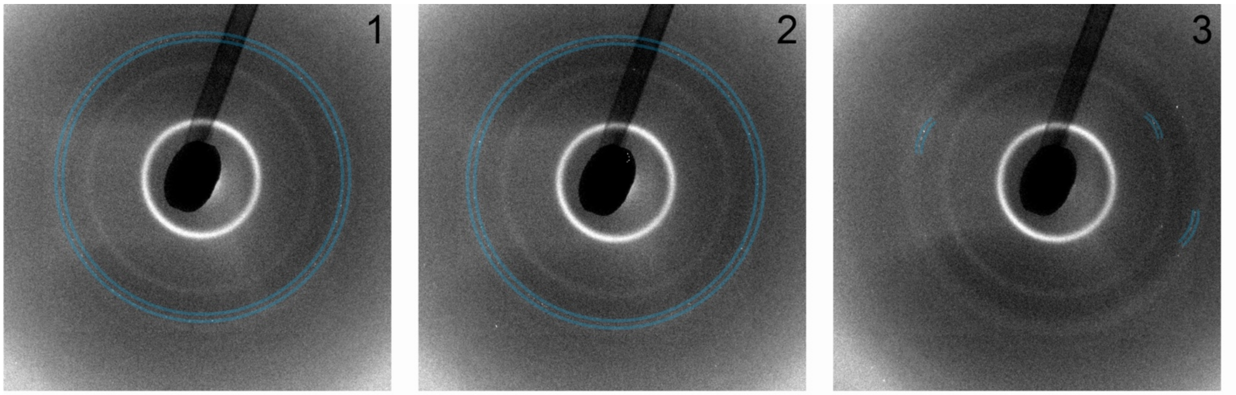

Supplementary Figure 16. Micro X-ray diffraction of a 6.9 SSL larva. Panels 1 and 2 show X-ray diffraction patterns from areas in the 1° interstripe and panel 3 from area adjacent to the 1° interstripe. A low angular distribution diffraction of the (002) plane (3) is visible adjacent to the first interstripe region (1 and 2).

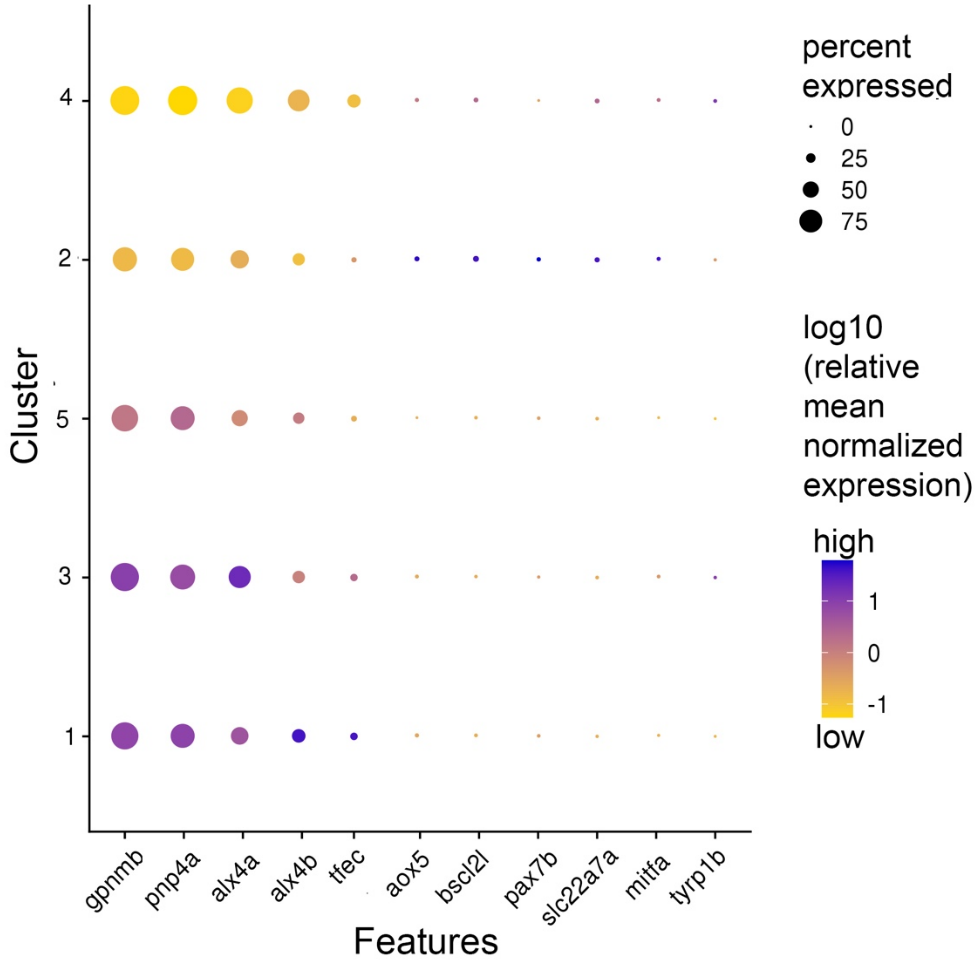

Supplementary Figure 17. Single-cell transcriptomic identification of cell types. Known cell-type marker genes for iridophores (*gpnmb*, *pnp4a*, *alx4a*, *alx4b*, *tfec*), xanthophores (*aox5*, *bscl2l*, *pax7b*, *slc22a7a*) and melanophores (*mitfa*, *tyrp1b*). Cells of clusters 1, 3 and 5 express high levels of known markers for iridophores, whereas cells of clusters 3 and 4 express low levels of these markers.

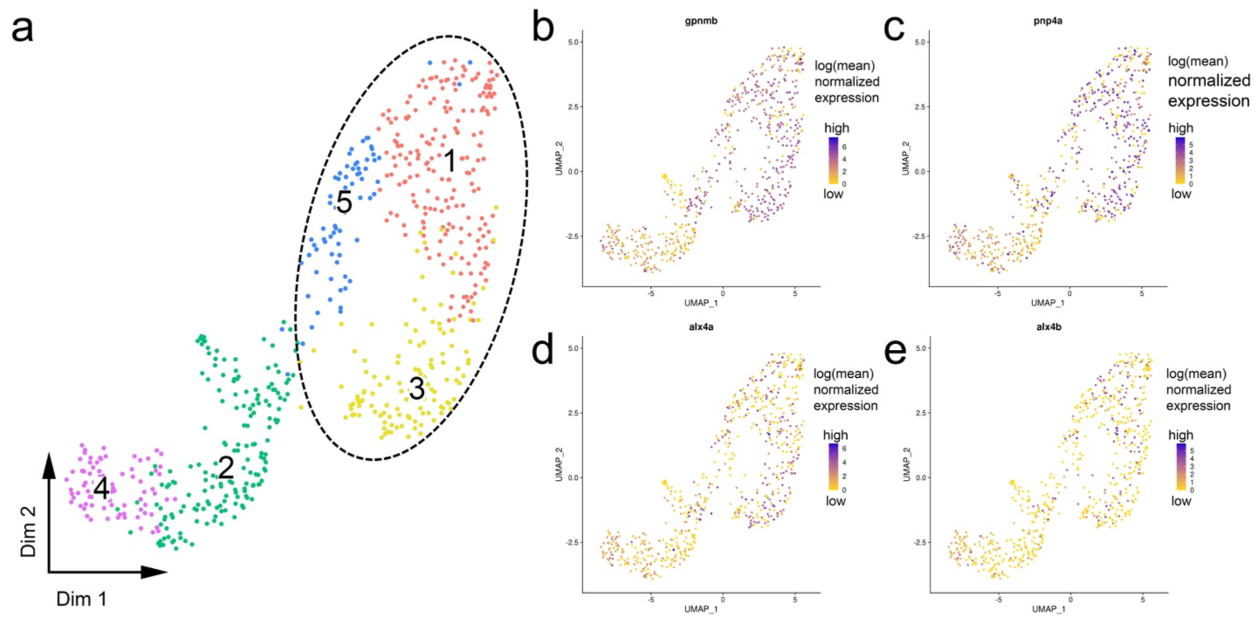

Supplementary Figure 18. Iridophores cluster specific markers. (a) Two-dimensional UMAP representation of the collected skin cell clusters (dashed ellipse marks iridophores**). (b-d)** UMAP plots of pigment cells colored by expression of iridophore cluster-enriched genes (b) *gpnmb* (c) *pnp4a* (d) *alx4a* (e) *alx4b.*

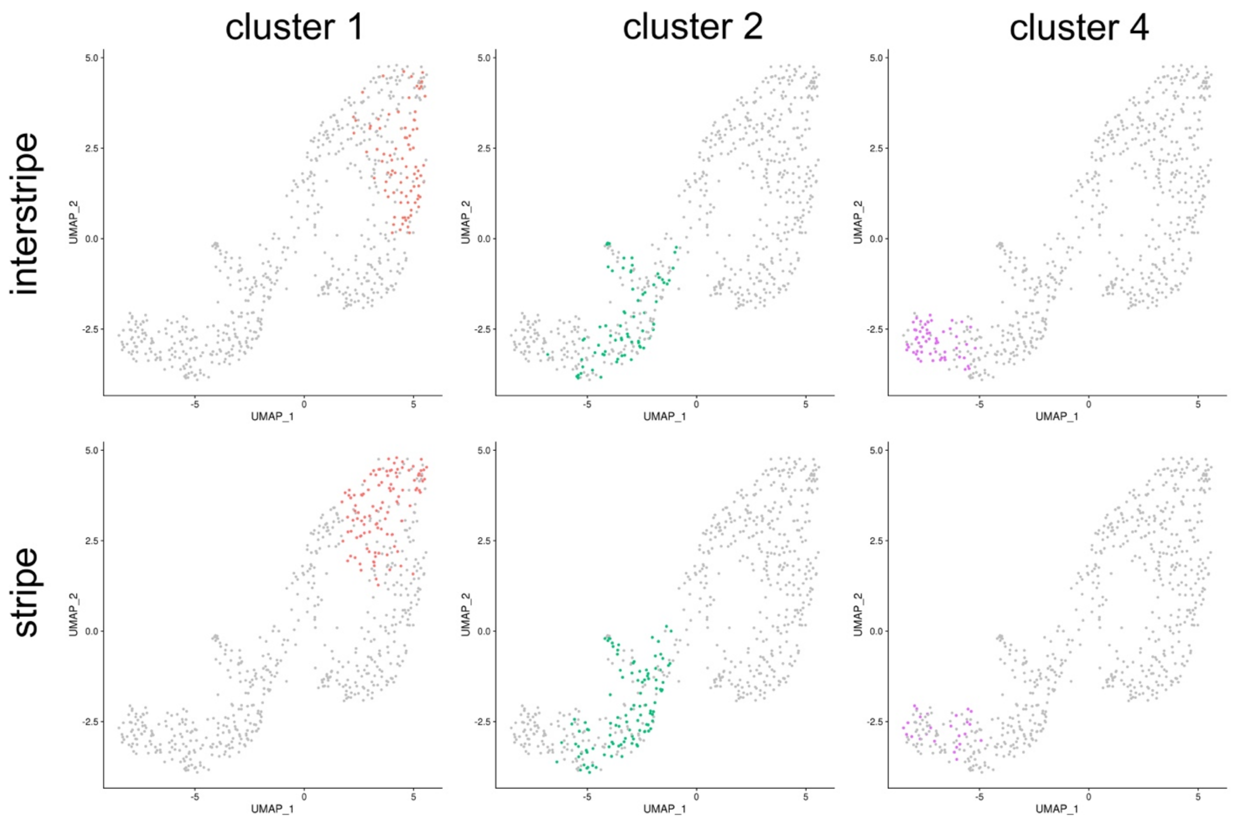

Supplementary Figure 19. Two-dimensional UMAP representation of the different clusters, where the anatomical origin (stripe or interstripe) of the different cells is marked in pseudo color (cluster 1 – red, cluster 2 – green, cluster 3 – purple). The cells of all three clusters shown, were shared between the stripe and the interstripe, with the following distribution: Cluster 1, 44% interstripe, 56% stripe. Cluster 2, 40% interstripe, 60% stripe. Cluster 4, 68% interstipe, 32% stripe.

a

b

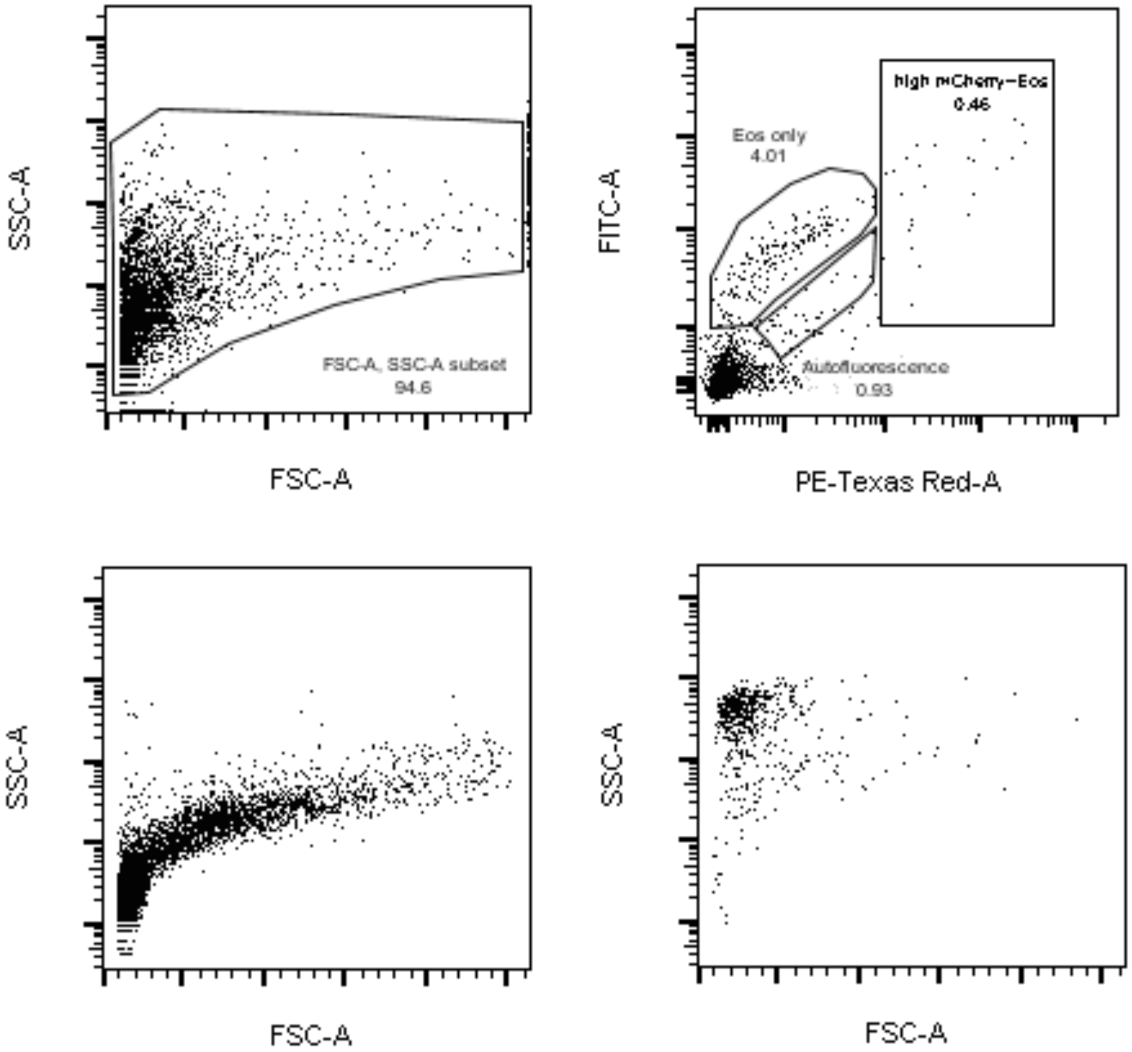

Supplementary Figure 20. The population of zebrafish skin iridophores was designated based on their FSC and SSC characteristics and back-gating on fluorescence. **(a)** The variation in the zebrafish skin cell size and internal complexity varied greatly such that only the extremely large FSC events were excluded with the initial scatter gate, 40k events. Control zebrafish skin samples were used to gate out the highly auto fluorescent cells among the members of this population. **(b)** Back-gating from the Eos (left) and mCherry (right) labeled cells were used to confirm the initial scatter gate, 450k events. Fluorescently labeled from either the stripe or interstripe skin samples were sorted separately. Single cells with high mCherry and Eos expression were collected. Five indexed 96 well plates were collected for each skin band.

Supplementary Table 1. Top 40 gene ontology biological process terms for the differentially expressed genes between clusters c2 and c4, determined using FishEnrichr[1, 2], where the p-value (p) is computed using the Fisher exact test and the Z-score (z) is computed by assessing the deviation from the expected rank. The combined score (c) is calculated as follows, $c = \log(p) \cdot z$

Index	Name	P-value	Adj P-value	Z-score	Combined Score
1	regulation of nucleobase-containing compound metabolic process	5.94E-06	4.28E-03	-2.73	32.84
2	response to purine-containing compound	1.02E-04	1.47E-02	-3.34	30.72
3	3'-UTR-mediated mRNA destabilization	3.39E-04	3.05E-02	-3.83	30.57
4	response to organophosphorus	1.02E-04	1.47E-02	-3.32	30.54
5	midbrain-hindbrain boundary morphogenesis	3.39E-04	3.05E-02	-3.68	29.42
6	mRNA destabilization	3.92E-05	1.41E-02	-2.87	29.15
7	cardiac cell development	7.83E-04	4.70E-02	-3.21	22.98
8	3'-UTR-mediated mRNA stabilization	5.58E-03	1.75E-01	-4.41	22.88
9	response to cAMP	4.61E-04	3.69E-02	-2.87	22.06
10	positive regulation of developmental process	1.77E-04	2.12E-02	-2.45	21.20
11	RNA destabilization	3.04E-03	1.22E-01	-3.65	21.15
12	mRNA stabilization	9.85E-04	5.46E-02	-2.69	18.62
13	regulation of RNA metabolic process	6.62E-05	1.47E-02	-1.75	16.84
14	positive regulation of cellular amide metabolic process	7.10E-03	1.97E-01	-3.38	16.73
15	positive regulation of mRNA processing	1.06E-02	2.14E-01	-3.65	16.58
16	retinoic acid metabolic process	8.42E-02	4.27E-01	-6.67	16.50
17	positive regulation of mRNA splicing, via spliceosome	1.06E-02	2.14E-01	-3.62	16.44
18	positive regulation of RNA splicing	1.26E-02	2.14E-01	-3.67	16.05
19	response to cytokine	1.48E-03	7.63E-02	-2.45	15.97
20	positive regulation of cellular protein metabolic process	8.79E-03	2.14E-01	-3.37	15.95
21	positive regulation of cell differentiation	7.83E-04	4.70E-02	-2.18	15.56
22	cellular response to hormone stimulus	2.20E-03	1.05E-01	-2.47	15.14
23	positive regulation of cell development	2.89E-03	1.22E-01	-2.54	14.83
24	vacuolar acidification	8.79E-03	2.14E-01	-3.05	14.43
25	sequestering of actin monomers	7.10E-03	1.97E-01	-2.80	13.85
26	wound healing	2.49E-03	1.12E-01	-2.30	13.78
27	intracellular pH reduction	1.26E-02	2.14E-01	-3.15	13.78
28	negative regulation of Ras protein signal transduction	7.10E-03	1.97E-01	-2.76	13.64
29	adenylate cyclase-inhibiting adrenergic receptor signaling pathway	8.42E-02	4.27E-01	-5.48	13.55
30	melanosome organization	1.71E-02	2.42E-01	-3.28	13.34
31	cardiac muscle cell differentiation	3.83E-03	1.31E-01	-2.37	13.21
32	negative regulation of neuron death	3.83E-03	1.31E-01	-2.27	12.64
33	negative regulation of hemopoiesis	8.42E-02	4.27E-01	-5.09	12.59
34	regulation of wound healing	8.42E-02	4.27E-01	-5.08	12.57
35	negative regulation of insulin receptor signaling pathway	1.26E-02	2.14E-01	-2.82	12.30
36	positive regulation of transporter activity	8.42E-02	4.27E-01	-4.91	12.15
37	regulation of protein polymerization	1.71E-02	2.42E-01	-2.91	11.84
38	regulation of myotube differentiation	9.75E-02	4.27E-01	-5.05	11.75
39	negative regulation of actin filament polymerization	1.71E-02	2.42E-01	-2.87	11.67
40	negative regulation of cellular response to insulin stimulus	1.26E-02	2.14E-01	-2.61	11.41

Supplementary Table 2. Top 40 gene ontology cellular component terms for the differentially expressed genes between clusters c2 and c4, determined using FishEnrichr[1, 2], where the p-value (p) is computed using the Fisher exact test and the Z-score (z) is computed by assessing the deviation from the expected rank. The combined score (c) is calculated as follows, $c = \log(p) \cdot z$.

Index	Name	P-value	Adj P-value	Z-score	Combined Score
1	proton-transporting V-type ATPase complex	8.79E-03	2.75E-01	-3.10	14.67
2	focal adhesion	1.61E-03	1.51E-01	-2.11	13.57
3	vacuolar proton-transporting V-type ATPase complex	1.48E-02	3.48E-01	-3.14	13.24
4	Rad51B-Rad51C-Rad51D-XRCC2 complex	9.75E-02	4.59E-01	-5.05	11.76
5	lipid droplet	3.07E-02	4.17E-01	-3.02	10.52
6	mitotic spindle pole	1.11E-01	4.59E-01	-4.36	9.60
7	mitochondrial proton-transporting ATP synthase complex, coupling factor F(o)	9.75E-02	4.59E-01	-3.62	8.43
8	cytosolic proteasome complex	1.24E-01	4.59E-01	-3.71	7.76
9	U2 snRNP	3.07E-02	4.17E-01	-2.19	7.62
10	contractile actin filament bundle	1.11E-01	4.59E-01	-3.02	6.64
11	mitochondrial proton-transporting ATP synthase complex	1.11E-01	4.59E-01	-2.89	6.36
12	COPII vesicle coat	1.36E-01	4.59E-01	-3.18	6.34
13	actomyosin	1.24E-01	4.59E-01	-2.95	6.16
14	exocytic vesicle	1.11E-01	4.59E-01	-2.65	5.83
15	secretory vesicle	1.11E-01	4.59E-01	-2.62	5.77
16	cytosolic ribosome	4.54E-02	4.59E-01	-1.83	5.67
17	cytosol	6.55E-03	2.75E-01	-1.12	5.64
18	caveola	1.74E-01	4.59E-01	-3.00	5.26
19	cytosolic small ribosomal subunit	1.01E-01	4.59E-01	-1.82	4.18
20	stress fiber	1.11E-01	4.59E-01	-1.88	4.15
21	pigment granule	1.74E-01	4.59E-01	-2.34	4.10
22	intermediate filament cytoskeleton	1.86E-01	4.59E-01	-2.38	4.01
23	chromatin	3.11E-02	4.17E-01	-1.10	3.81
24	replication fork	2.21E-01	4.59E-01	-2.46	3.72
25	mitochondrial respiratory chain complex IV	2.09E-01	4.59E-01	-2.28	3.57
26	U1 snRNP	2.43E-01	4.59E-01	-2.46	3.47
27	cytosolic part	8.10E-02	4.59E-01	-1.36	3.43
28	prespliceosome	2.09E-01	4.59E-01	-2.13	3.33
29	spliceosomal snRNP complex	1.34E-01	4.59E-01	-1.57	3.15
30	transcription factor TFIID complex	2.65E-01	4.70E-01	-2.31	3.07
31	melanosome	1.74E-01	4.59E-01	-1.75	3.06
32	bicellular tight junction	2.32E-01	4.59E-01	-2.05	3.00
33	nuclear chromatin	6.00E-02	4.59E-01	-1.06	2.99
34	small ribosomal subunit	1.24E-01	4.59E-01	-1.39	2.90
35	intermediate filament	2.21E-01	4.59E-01	-1.92	2.90
36	U2-type prespliceosome	2.09E-01	4.59E-01	-1.83	2.87
37	perinuclear region of cytoplasm	1.49E-01	4.59E-01	-1.50	2.85
38	nucleus	6.77E-02	4.59E-01	-1.00	2.69
39	intercalated disc	1.86E-01	4.59E-01	-1.58	2.66
40	autophagosome membrane	2.43E-01	4.59E-01	-1.83	2.59

References

1. Chen EY*, et al.* Enrichr: interactive and collaborative HTML5 gene list enrichment analysis tool. *BMC Bioinformatics* **14**, 128 (2013).

2. Kuleshov MV*, et al.* Enrichr: a comprehensive gene set enrichment analysis web server 2016 update. *Nucleic Acids Res* **44**, W90-97 (2016).
