# Supplementary material for: In situ differentiation of iridophore crystallotypes underlies zebrafish stripe patterning
Source: Nat Commun. 2020 Dec 15;11:6391. doi: 10.1038/s41467-020-20088-1 (PMC7738553; doi:10.1038/s41467-020-20088-1)
Supplement: Supplementary file 12 — Description of Additional Supplementary Files [file 41467_2020_20088_MOESM12_ESM.docx]

Description of additional supplementary information

Title: Supplementary Movie S1.

Description: Proliferation of densely arranged pnp4+ cells. Region shown corresponds to that described in Figure 2A with arrowheads illustrating cell division both at the edge of the interstripe and within it. Regions shown in Supplementary Movies S1–S5 are selected from larger fields of view to highlight specific behaviors.

Title: Supplementary Movie S2.

Description: Proliferation and differentiation of pnp4+ cells adjacent to interstripe. Region shown corresponds to that of Figure 2B, with orange arrowheads indicating proliferative cell and daughters.

Title: Supplementary Movie S3.

Description: Proliferation and differentiation of loosely arranged pnp4+ cells within the prospective stripe. Region shown includes that in Figure 2C. Orange arrowheads indicate proliferative cell and daughters, purple arrowhead indicates cell newly expressing pnp4a:mem-Cherry.

Title: Supplementary Movie S4.

Description: Proliferation and differentiation of loosely arranged pnp4+ cells within the prospective stripe. Region shown corresponds to that of Figure 2D. Arrowheads indicate proliferating cells and daughters except for purple arrowhead that marks a ventral cell newly acquiring pnp4a:mem-Cherry expression.

Title: Supplementary Movie S5.

Description: Proliferation and migration of pnp4+ cells within stripe. Region corresponds to Figure 2E, with arrowheads identifying a cell that divides into daughter cells that become widely separated after one migrates ventrally.

Title: Supplementary Movie S6.

Description: Iridophore patterning from anterior to posterior. Movie shows a typical individual, imaged daily from stages of primary interstripe development through stripe formation and interstripe reiteration. Individual corresponds to that shown in Extended Data Figure 4a,b. Images are rescaled and aligned to control for growth.

Title: Supplementary Movie S7.

Description: Iridophore patterning from anterior to posterior. Movie shows an individual that represents a minority of instances, with transiently dense clusters of iridophores in the prospective dorsal stripe, as well as a patche of densely arranged iridophores that splits ventrally between primary and second interstripe. Movie corresponds to individual shown in Extended Data Figure 4c,d. Images are rescaled and aligned to control for growth.

Title: Supplementary Movie S8.

Description: The response of the stripes pattern to NE. Movie shows the stripe pattern response of an adult zebrafish to NE. Minutes after administrating NE the contrast between the dark blue stripe and the yellow interstripe is drastically diminished as the pigment granules within the melanophores and xanthophores are aggregated in the cells center and the stripe iridophores changed their color from blue to yellow.

Title: Supplementary Table 1.

Description: The metadata of sc-RNAseq clustering used to make figure 5b. With per cluster information on: number of cells, percent of the cells expressing each gene, mean expression value, Standard deviation of expression.

Title: Supplementary Table 2.

Description: A list of dysregulated features between cluster 3c and 5c, which was used to make Figure 5d
