# Supplementary material for: In situ differentiation of iridophore crystallotypes underlies zebrafish stripe patterning
Source: Nat Commun. 2020 Dec 15;11:6391. doi: 10.1038/s41467-020-20088-1 (PMC7738553; doi:10.1038/s41467-020-20088-1)
Supplement: Supplementary file 13 — Reporting Summary [file 41467_2020_20088_MOESM13_ESM.pdf]

nature research

Corresponding author(s): Jennifer Lippincott Schwartz

Last updated by author(s): Nov 3, 2020

Reporting Summary

Nature Research wishes to improve the reproducibility of the work that we publish. This form provides structure for consistency and transparency in reporting. For further information on Nature Research policies, see our Editorial Policies and the Editorial Policy Checklist.

Statistics

For all statistical analyses, confirm that the following items are present in the figure legend, table legend, main text, or Methods section.

n/a	Confirmed	
☐	☒	The exact sample size (*n*) for each experimental group/condition, given as a discrete number and unit of measurement
☐	☒	A statement on whether measurements were taken from distinct samples or whether the same sample was measured repeatedly
☐	☒	The statistical test(s) used AND whether they are one- or two-sided<br>*Only common tests should be described solely by name; describe more complex techniques in the Methods section.*
☐	☒	A description of all covariates tested
☐	☒	A description of any assumptions or corrections, such as tests of normality and adjustment for multiple comparisons
☐	☒	A full description of the statistical parameters including central tendency (e.g. means) or other basic estimates (e.g. regression coefficient) AND variation (e.g. standard deviation) or associated estimates of uncertainty (e.g. confidence intervals)
☐	☒	For null hypothesis testing, the test statistic (e.g. F, t, r) with confidence intervals, effect sizes, degrees of freedom and P value noted<br>*Give P values as exact values whenever suitable.*
☐	☒	For Bayesian analysis, information on the choice of priors and Markov chain Monte Carlo settings
☐	☒	For hierarchical and complex designs, identification of the appropriate level for tests and full reporting of outcomes
☐	☒	Estimates of effect sizes (e.g. Cohen's *d*, Pearson's *r*), indicating how they were calculated

Our web collection on statistics for biologists contains articles on many of the points above.

Software and code

Policy information about availability of computer code

Data collection	ZEN (v2.3 blue edition, v2.1 black edition, for florescence and optical microscopy), Python (v2.7, for X-ray diffraction), PARISS custom software (for hyper-spectral imaging), FEI, serialEM (v3.8, for TEM), ZEISS ULTRA SEM software (V5.06.for SEM), BD FACSDiva software (v7.1, for FACS), NextSeq System Suite (v2.3for scRNA-Seq). Cutadapt (v2.9,sequensing).
Data analysis	R (v3.6,for scRNA-Seq Analysis), ImageJ (v1.52j, for time lapse imaging and analyses), Matlab (vr2019a,for optical measurements data analysis and graph plotting), Fit2D (v18,for X-ray diffraction analysis), Lab Spec 6 (for micro-Raman data analysis), Illustrator (v2020), and Photoshop (cc2019) (for preparing figures), Prism 8 (for graph plotting), JMP v14 (for statistical analysis)

For manuscripts utilizing custom algorithms or software that are central to the research but not yet described in published literature, software must be made available to editors and reviewers. We strongly encourage code deposition in a community repository (e.g. GitHub). See the Nature Research guidelines for submitting code & software for further information.

Data

Policy information about availability of data

All manuscripts must include a data availability statement. This statement should provide the following information, where applicable:

- Accession codes, unique identifiers, or web links for publicly available datasets
- A list of figures that have associated raw data
- A description of any restrictions on data availability

https://www.ncbi.nlm.nih.gov/geo/query/acc.cgi?acc=GSE144734

Field-specific reporting

Please select the one below that is the best fit for your research. If you are not sure, read the appropriate sections before making your selection.

☒ Life sciences ☐ Behavioural & social sciences ☐ Ecological, evolutionary & environmental sciences

For a reference copy of the document with all sections, see nature.com/documents/nr-reporting-summary-flat.pdf

Life sciences study design

All studies must disclose on these points even when the disclosure is negative.

Sample size	Sample sizes were determined a priori according to prior experience detecting effect sizes likely to be biologically relevant and limitations of obtaining and analyzing experimental material. Statistical analyses were performed only after all data had been collected. Formal power analyses were not performed.
Data exclusions	No data were excluded
Replication	Analyses of cell behaviors were replicated across animals; differences in outcomes were not evident.
Randomization	Both wild type and transgenic fish were randomly allocated for the experiments.
Blinding	For crystal size and distribution measurements, investigators were blind to the sampled group.<br>Blinding was not possible for time-lapse, cell fate, optical studies or X-ray studies as pattern and cell phenotype is visible.

Reporting for specific materials, systems and methods

We require information from authors about some types of materials, experimental systems and methods used in many studies. Here, indicate whether each material, system or method listed is relevant to your study. If you are not sure if a list item applies to your research, read the appropriate section before selecting a response.

Materials & experimental systems

n/a	Involved in the study	
☒	☐	Antibodies
☒	☐	Eukaryotic cell lines
☒	☐	Palaeontology and archaeology
☐	☒	Animals and other organisms
☒	☐	Human research participants
☒	☐	Clinical data
☒	☐	Dual use research of concern

Methods

n/a	Involved in the study	
☒	☐	ChIP-seq
☐	☒	Flow cytometry
☒	☐	MRI-based neuroimaging

Animals and other organisms

Policy information about studies involving animals; ARRIVE guidelines recommended for reporting animal research

Laboratory animals	Zebrafish (danio rerio), both males and females, both adults (6 months to 12 months) and juvenile (14 dpf to 45 dpf). For the electron microscopy and X-ray diffraction 6 -12 months or 14 - 45 dpf fish were used. For the experiments following the development of juvenile fish (light microscopy and X ray diffraction), 14 dpf to 45 dpf dish were used.
Wild animals	No wild animals were used in these study
Field-collected samples	No field-collected samples were used in these study
Ethics oversight	HHMI-JFRC ethical committee via animal Care and Use protocol 16-137, and UVA IACUC protocol 4170.

Note that full information on the approval of the study protocol must also be provided in the manuscript.

Flow Cytometry

Plots

Confirm that:

☒ The axis labels state the marker and fluorochrome used (e.g. CD4-FITC).

☒ The axis scales are clearly visible. Include numbers along axes only for bottom left plot of group (a 'group' is an analysis of identical markers).

☒ All plots are contour plots with outliers or pseudocolor plots.

☒ A numerical value for number of cells or percentage (with statistics) is provided.

Methodology

Sample preparation	Fish were euthanized in MS222(E10521, Sigma-Aldrich) and the stripe and interstiped regions were micro-dissected from fish expressing both pnp4a:mem-mCherry and pnp4a:nlsEos. Stripe and interstipe regions were enzymatically dissociated separately with Liberase (0.25 mg/ml in dPBS, (LIBDL-RO, Roche) at 25°C for 15 min followed by manual trituration with increasingly narrower flame polished glass pipette for 3 min at a time for three times. Cells suspensions were then filtered through a 70 µm Nylon cell strainer to obtain a single cell suspension. Liberated cells were re-suspended in 1% BSA (A2153, Sigma-Aldrich) / 5% FBS (F2442, Sigma-Aldrich) in dPBS before FACS purification. This was done for samples collected from four different fish that were processed in two different cycles, combining skin samples from two different fish for each cycle.
Instrument	The flow cytometry experiments were performed on a BD FACSAria II SORP sorter (BD Biosciences, San Jose, CA, USA)
Software	Data collection was performed using BD FACSDiva software (BD Biosciences)
Cell population abundance	Double positive cells (of pnp4:mem-Cherry+ ,pnp4a:nucEos+) cells, were 0.3%-1% of the total population, which is within the standards in the field. Regarding post sorting population, based on the sc-RNA sequencing we identified 63% of the cells collected as fully differentiated iridophores.
Gating strategy	. A 637 nm and a 488 nm laser were utilized for fluorophore-excitation and a 100-µm nozzle was used to generate single droplets under 20PSI sheath pressure. The applied settings were as follows: forward light scatter (FSC) detector photomultiplier tube (PMT) gain setting = 80 V with a 1.5 neutral density filter; side light scatter (SSC) detector PMT gain setting = 90 V; FSC threshold = 10,000; PE-Texas Red (PE TX Red) channel PMT gain setting = 280 V; fluorescein isothiocyanate (FITC) channel PMT gain setting = 280 V. Sample dilution and flow rate were adjusted to optimal event recordings for 96 well plate single cell sorts (below 500 processed events per sec). The population of zebrafish skin iridophores was designated based on their FSC and SSC characteristics and back-gating on fluorescence. Control zebrafish skin samples were used to gate out the highly auto fluorescent cells among the members of this population. Fluorescently labeled from either the stripe or interstripe skin samples were sorted separately. An exemplification the gating strategy is provided in Supplementary Figure 20.

☒ Tick this box to confirm that a figure exemplifying the gating strategy is provided in the Supplementary Information.
